# Potent Dual Polymerase/Exonuclease Inhibitory Activities of Antioxidant Aminothiadiazoles Against the COVID-19 Omicron Virus: A Promising In Silico/In Vitro Repositioning Research Study

**DOI:** 10.1007/s12033-022-00551-8

**Published:** 2023-01-24

**Authors:** Amgad M. Rabie, Wafa A. Eltayb

**Affiliations:** 1 Dr. Amgad Rabie’s Research Lab. for Drug Discovery (DARLD), Mansoura City, Mansoura, 35511 Dakahlia Governorate Egypt; 2 Head of Drug Discovery & Clinical Research Department, Dikernis General Hospital (DGH), Magliss El-Madina Street, Dikernis City, Dikernis, 35744 Dakahlia Governorate Egypt; 3 Biotechnology Department, Faculty of Science and Technology, Shendi University, Shendi, Nher Anile Sudan

**Keywords:** Anti-Omicron Agent, Anti-COVID-19 Drug, Coronaviral-2 RNA-dependent RNA Polymerase (RdRp), SARS-CoV-2 Proofreading 3′-to-5′ Exoribonuclease (ExoN), 2-Amino-1,3,4-thiadiazole Derivative, ChloViD2022

## Abstract

Recently, natural and synthetic nitrogenous heterocyclic antivirals topped the scene as first choices for the treatment of the severe acute respiratory syndrome coronavirus 2 (SARS-CoV-2) infections and their accompanying disease, the coronavirus disease 2019 (COVID-19). Meanwhile, the mysterious evolution of a new strain of SARS-CoV-2, the Omicron variant and its sublineages, caused a new defiance in the continual COVID-19 battle. Hitting the two principal coronaviral-2 multiplication enzymes RNA-dependent RNA polymerase (RdRp) and 3′-to-5′ exoribonuclease (ExoN) synchronously using the same ligand is a highly effective novel dual pathway to hinder SARS-CoV-2 reproduction and stop COVID-19 progression irrespective of the SARS-CoV-2 variant type since RdRps and ExoNs are widely conserved among all SARS-CoV-2 strains. Herein, the present computational/biological study screened our previous small libraries of nitrogenous heterocyclic compounds, searching for the most ideal drug candidates predictably able to efficiently act through this double approach. Theoretical filtration gave rise to three promising antioxidant nitrogenous heterocyclic compounds of the 1,3,4-thiadiazole type, which are CoViTris2022, Taroxaz-26, and ChloViD2022. Further experimental evaluation proved for the first time, utilizing the in vitro anti-RdRp/ExoN and anti-SARS-CoV-2 bioassays, that ChloViD2022, CoViTris2022, and Taroxaz-26 could effectively inhibit the replication of the new virulent strains of SARS-CoV-2 with extremely minute in vitro anti-RdRp and anti-SARS-CoV-2 EC_50_ values of 0.17 and 0.41 μM for ChloViD2022, 0.21 and 0.69 μM for CoViTris2022, and 0.23 and 0.73 μM for Taroxaz-26, respectively, transcending the anti-COVID-19 drug molnupiravir. The preliminary in silico outcomes greatly supported these biochemical results, proposing that the three molecules potently strike the key catalytic pockets of the SARS-CoV-2 (Omicron variant) RdRp’s and ExoN’s vital active sites. Moreover, the idealistic pharmacophoric hallmarks of CoViTris2022, Taroxaz-26, and ChloViD2022 molecules relatively make them typical dual-action inhibitors of SARS-CoV-2 replication and proofreading, with their highly flexible structures open for various kinds of chemical derivatization. To cut it short, the present pivotal findings of this comprehensive work disclosed the promising repositioning potentials of the three 2-aminothiadiazoles, CoViTris2022, Taroxaz-26, and ChloViD2022, to successfully interfere with the crucial biological interactions of the coronaviral-2 polymerase/exoribonuclease with the four principal RNA nucleotides, and, as a result, cure COVID-19 infection, encouraging us to rapidly start the three drugs’ broad preclinical/clinical anti-COVID-19 evaluations.

**Graphical Abstract:**

Dual SARS-CoV-2 polymerase (RdRp) and exoribonuclease (ExoN) inhibition via nucleoside mimicry is a very effective novel approach for COVID-19 infection therapy. Hydroxylated nitrogenous heterocyclic compounds are currently considered first choices in COVID-19 therapy. Extensive computational investigations disclosed three synthetic 5-substituted-2-amino-1,3,4-thiadiazoles, CoViTris2022, Taroxaz-26, and ChloViD2022, with ideal anti-RdRp/ExoN features. ChloViD2022 was ranked the top among the three NAs, with biochemical anti-RdRp EC_50_ value of 0.17 μM. ChloViD2022 accordingly displayed excellent anti-SARS-CoV-2 EC_50_ value of 0.41 μM against the Omicron variant.

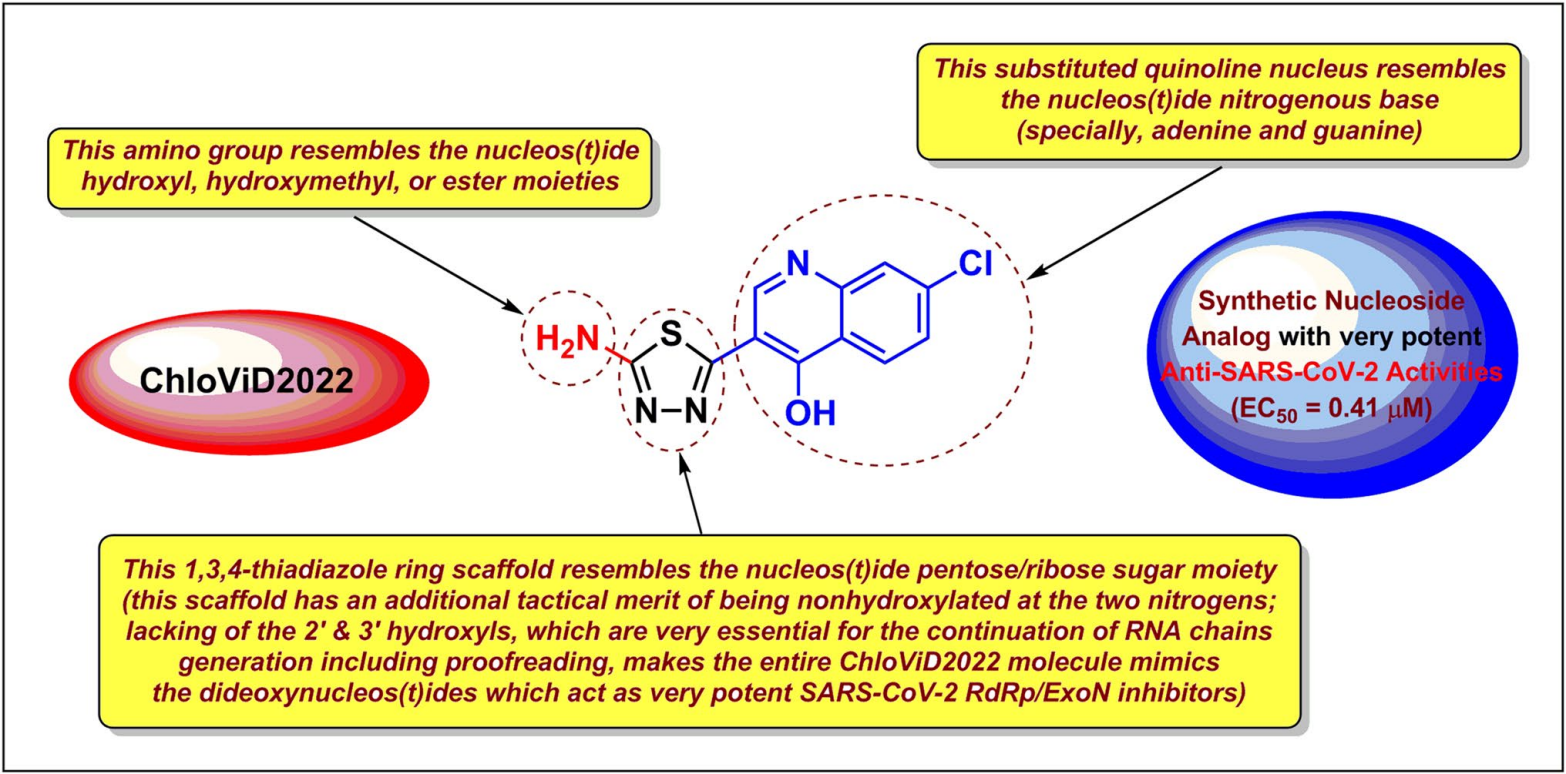

**Supplementary Information:**

The online version contains supplementary material available at 10.1007/s12033-022-00551-8.

## Introduction

In the preceding 2 years (2020–2021) since the severe acute respiratory syndrome coronavirus 2 (SARS-CoV-2) blazed across the globe, we and our multinational multidisciplinary research team have been in our respective laboratories day and night scrutinizing coronavirus disease 2019 (COVID-19) cases among people of different sexes/ages/races/cultures, designing new drugs against the virus, repositioning recognized remedies against the disease, and exchanging our relevant opinions and insights with colleagues in Egypt, Arab countries, China, the USA, and many other countries. There are three major requirements that have yet to be adequately met for efficient and successful management/treatment of COVID-19 conditions: (1) potent antiviral medications that significantly restrict SARS-CoV-2 transmission, cell entry, replication, and pathogenicity, (2) medications that attenuate the acute nonproductive immune responses and thus considerably decrease end-organ damage, and (3) medications that have a strong antifibrotic action in patients with acute respiratory distress syndrome (ARDS) and thus combat the long-term sequelae of this irritating disease [1–7]. Compounds and drugs that act to satisfy primarily the first need of the previous three ones are comparatively few to date. Mainly, nucleoside analogs (NAs) and polyphenolics (PPhs) possessing nitrogenous heterocyclic rings in their scaffolds have shown significant successful lead and advancement as SARS-CoV-2 inhibitors and virucides [8–19]. Naturally, NAs and PPhs are two of the most biocompatible and tolerated classes in human bodies among all chemical structures [16–20]. Some new and repurposed efficacious nucleoside-like compounds are nowadays under wide investigations to be biologically and clinically assessed as effective prospective anti-COVID-19 medicines, e.g., nirmatrelvir, molnupiravir, remdesivir, GS-441524, GS-443902, cordycepin, didanosine, and favipiravir, but only the first three examples reached to the clinical use stage successfully to date (only against the mild-to-moderate COVID-19 cases) [8–15].

The mysterious SARS-CoV-2 Omicron variant, also known as B.1.1.529 (or BA), first began its tear around the world late 2021 and now has more than three sisters of BA sublineages, e.g., BA.1, BA.2, and BA.3 [21]. South African scientists reported the new variant on November 24, 2021, immediately after its first appearance [21]. As of January 7, the World Health Organization (WHO) reports that this highly infectious and virulent variant had been detected in more than 150 countries [21]. Omicron variant has at least thirty six new mutations in its spike (S) proteins [22]. Being unfixed and changeable day by day from one strain to the newer, S protein is not that attractive target for designing new remedies against SARS-CoV-2 strains. While, on the other hand, targeting the universal fixed proteins among all strains, e.g., SARS-CoV-2 replication RNA-dependent RNA polymerase (RdRp) and proofreading 3′-to-5′ exoribonuclease (ExoN) enzymes, through properly repurposing known compounds is much more effective and time-saving approach in this battle, even against the expectedly coming resistant coronaviral-2 strains. Moreover, medications targeting the S protein have only one chance to fight the coronaviral-2 infection, since after passage of any viral particles inside the host body (or if these therapies were taken after the occurrence of the infection) there will not be any further abilities of these therapies to stop virus propagation and infection. Unlike therapies targeting the replication and proofreading enzymes, which have unlimited number of continuous chances to fight the virus and its successors and prevent their further multiplication throughout the entire human body (even if these therapies were taken after the occurrence of the infection). In the first months of 2022, we as a multidisciplinary team continued our scientific journey and worked around the clock to discover efficient anti-SARS-CoV-2-Omicron-variant drug candidates.

**Fig. 1:**
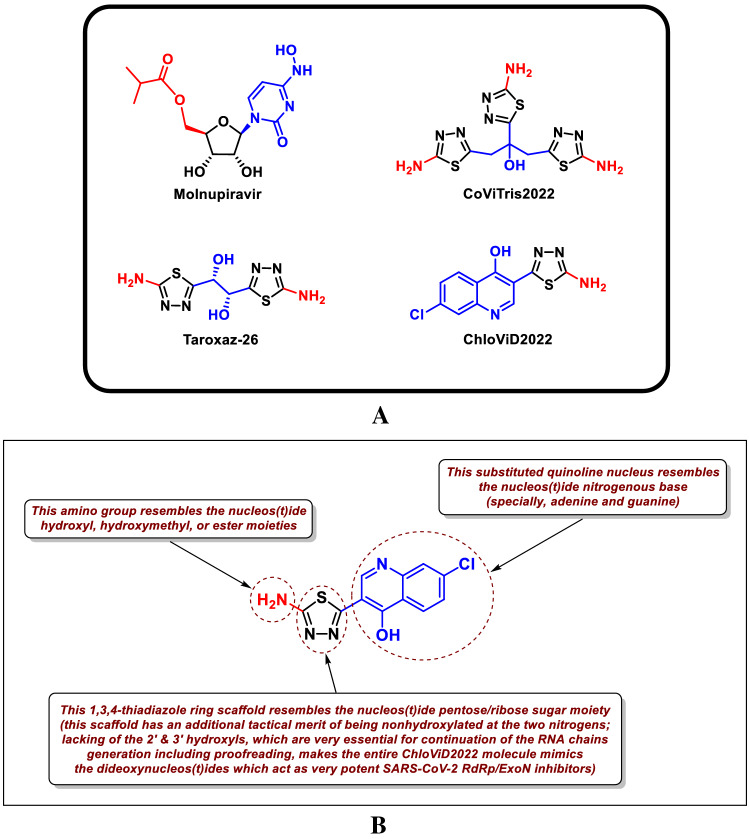
**A** Chemical structures of the reference anti-SARS-CoV-2 drug molnupiravir and the investigated three aminothiadiazoles, CoViTris2022, Taroxaz-26, and ChloViD2022. **B** Rational design of 5-substituted-2-amino-1,3,4-thiadiazoles, e.g., ChloViD2022 molecule as a displayed ideal example, as potential dual-action inhibitors of SARS-CoV-2 RdRp/ExoN acting through nucleoside mimicry strategy

Tactical nucleoside analogism is among the convenient therapeutic choices in medicinal chemists’ minds to resist and stop the rapid multiplication of SARS-CoV-2 particles inside the human body [9–15, 20]. In this SARS-CoV-2/COVID-19 therapeutic tactic, we make use of the close similarity of the used nucleoside/nucleotide analog with the normal natural nucleosides and nucleotides to misguide and deceive the SARS-CoV-2 RdRp (the nonstructural protein complex 12/7/8 or nsp12-nsp7-nsp8) and ExoN (the nonstructural protein complex 14/10 or nsp14-nsp10) enzymes [20]. Nsp12-nsp7-nsp8 and nsp14-nsp10 protein complexes are very indispensable enzymes in the replication/proofreading of the coronaviral-2 genome and, thus, their strong inhibition will significantly block the replication of SARS-CoV-2 particles. Nucleoside-like agents confuse both RdRp and ExoN enzymes through full incorporation in the viral RNA genetic strands in place of the correct endogenous nucleosides/nucleotides, giving rise to repeated excessive ambiguous coding and premature termination of RNA synthesis with the formation of vague RNA strands at the end; these faulty strands represent abnormal noninfectious and inactive particles, hence there would not be any further multiplication of the virus [13, 14, 20]. Most of the aforementioned anti-COVID-19 agents, e.g., molnupiravir and its active metabolite β-D-*N*^4^-hydroxycytidine (NHC) (Fig. 1A), draw on this highly effective mechanism in their inhibitory/blocking activities on the SARS-CoV-2 particles [9–12]. With the progressive evolution of more infectious variants of SARS-CoV-2, designing and synthesizing more potent and broad-spectrum anti-SARS-CoV-2 drugs became a must.

Nitrogenous heterocyclic aromatic compounds having the structural features of both NAs and PPhs like polyhydroxyphenyl-substituted 1,3,4-oxadiazoles proved their abilities to effectively hinder SARS-CoV-2 replication [16–19]. Analogously, 2-amino-1,3,4-thiadiazoles having hydroxyl group(s) in their structures are expected to have similar potent anti-COVID-19 activities like these polyhydroxyphenolic oxadiazoles. This logic expectation rendered us computationally screening our own small library of previously synthesized 2-amino-1,3,4-thiadiazole derivatives against SARS-CoV-2 targets to search for potential anti-COVID-19 agents [23]. Based on this in silico filtration and evaluation, the three compounds with the best results, CoViTris2022 (1,2,3-tris(5-amino-1,3,4-thiadiazol-2-yl)propan-2-ol; the number “2022” refers to the current work year), Taroxaz-26 ((1*R*,2*R*)-1,2-bis(5-amino-1,3,4-thiadiazol-2-yl)ethane-1,2-diol; the number “26” refers to the 2 oxygen and 6 nitrogen atoms, respectively, present in the molecule), and ChloViD2022 (3-(5-amino-1,3,4-thiadiazol-2-yl)-7-chloroquinolin-4-ol; the number “2022” refers to the current work year), were selected for the current work (Fig. 1A). The three molecules have three, two, and one 1,3,4-thiadiazole ring(s), respectively. CoViTris2022, Taroxaz-26, and ChloViD2022 were easily synthesized via oxidative cyclocondensation of thiosemicarbazide with their corresponding carboxylic acids (anhydrous citric acid, (2*R*,3*R*)-(+)-tartaric acid, and 7-chloro-4-hydroxy-3-quinolinecarboxylic acid, respectively) under conventional reflux heating or microwave irradiation heating [23]. The three 1,3,4-thiadiazoles and their mercapto derivatives possess several promising antioxidant/anticancer properties [23]. The structural resemblance and analogism of these three simple molecules, especially ChloViD2022 molecule, with the SARS-CoV-2 RNA nucloes(t)ides is expected to significantly help in deceiving and/or inhibiting the principal SARS-CoV-2 replication enzymes, such as RdRp and ExoN (Fig. 1B).

In the current research work, we have explored the combined inhibitory activities of these 2-amino-1,3,4-thiadiazoles on both SARS-CoV-2 RdRp and ExoN enzymes as a novel effective approach to double combat COVID-19 infections [24]. Theoretically among the three compounds, ChloViD2022 has the most typical chemical structure to become a candidate synthetic nucleoside analog or, more accurately, dideoxynucleoside analog with significant potentials to act as anti-SARS-CoV-2 RdRp/ExoN (as illustrated in Fig. 1B) [13, 14]. Computation-based molecular docking speculatively disclosed that the three compounds CoViTris2022, Taroxaz-26, and ChloViD2022 showed very good binding free energies with both enzymes, SARS-CoV-2 RdRp and ExoN, compared to those of the positive control (reference) molnupiravir with the same two enzymes (these very good computational binding affinities values were practically proven later based on the biological evaluation results which revealed the very small corresponding EC_50_/EC_90_ and Ki “inhibitory constant” values that are well correlated with the computational values). Molecular docking and dynamics simulations studies of the chosen three compounds disclosed the relative ideality of their most molecular modeling outputs and parameters values (including, e.g., interactions profiles, energy values, and final positions in the proteins) when compared with the anti-COVID-19 drug molnupiravir. The three molecules seem to hit the catalytic active sites of both coronaviral-2 multiplication enzymes with the formation of significantly stable complexes having relatively high net negative binding free energies in comparison to molnupiravir. Biological evaluations of the three thiadiazoles against both coronaviral-2 RdRp and ExoN proteins and against the entire SARS-CoV-2 Omicron-variant particles demonstrated nearly the same interesting potential of these compounds to act as anti-COVID-19 agents. There are slight differences in potencies among the three synthetic molecules, with ChloViD2022 being the superior compound in most computational and biochemical anti-SARS-CoV-2 examinations (with relatively significant target specificities/selectivities).

Based on these current results and previous data [25–28], we can conclude that, first, ChloViD2022, CoViTris2022, and Taroxaz-26, respectively, can be further in vivo and clinically investigated for repurposing against COVID-19 and, second, the expected potent clinical inhibitory effects of the three compounds against SARS-CoV-2 replication may be principally attributed to the triple synergistic inhibitory activities against the three enzymes RdRp, ExoN, and adenosine kinase (ADK), i.e., may be closely related to RdRp, ExoN, and ADK inhibitory activities of ChloViD2022, CoViTris2022, and Taroxaz-26. The possible SARS-CoV-2 RNA mutagenicity of the three ligands via nucleoside mimicry mode of action and combination into the new coronaviral-2 RNA strands needs also to be extensively and clinically explored. As an important step in our journey to try repositioning these three compounds against COVID-19, the pharmacokinetics of these molecules should be significantly put into consideration, because tissue distributions of these potential anticoronaviral-2 agents will undoubtedly affect their total capabilities of reducing viral loads of SARS-CoV-2 particles in COVID-19 medication [29]. The possibility of pharmaceutically formulating these promising aminothiadiazoles as rapid-action nasal/oral anti-COVID-19 sprays/drops should also be considered if they successfully passed the in vivo/clinical investigations with highly significant outcomes.

## Materials and Methods

### In Silico Computational Evaluation

#### Preparation of Targeted Coronaviral-2 Proteins

The 3D structures of the targeted SARS-CoV-2 RdRp and ExoN proteins were obtained from the RCSB Protein Data Bank (PDB) with PDB identification codes 7BV2 and 7MC6, respectively. Both enzymatic proteins were obtained in the complex forms with their protein cofactors (i.e., were obtained cocrystallized in the nsp12-nsp7-nsp8 and nsp14-nsp10 complex forms, respectively) to increase nature simulation. Proteins were viewed through Pymol Molecular Graphic Visualizer software 2.4 and their predetected active site residues (with their closest neighboring residues) were then checked for complete presence and correctness. The catalytic active site residues highlighted through Pymol software were noted for the next in silico studies.

#### Selection and Preparation of Target Aminothiadiazole Ligands

To choose the best 2-amino-1,3,4-thiadiazoles for the current study, a primary validated virtual screening of our small library, which consists of thirteen 5-substituted-2-amino-1,3,4-thiadiazoles [23], was done against SARS-CoV-2 RdRp and ExoN proteins using the Molecular Operating Environment (MOE) platform (Chemical Computing Group), following the known remdesivir protocol with its parameters and docking cavities in both enzymes. The three 2-amino-1,3,4-thiadiazole derivatives with the top collective results as the best hitting candidates of both proteins (ChloViD2022, CoViTris2022, and Taroxaz-26, respectively) were selected to continue the long procession of the present research study. After this accurate screening, an extensive literature survey was also performed for the exploration of the potentials of the chosen three 2-amino-1,3,4-thiadiazoles to be antivirals. The chemical structures of the selected 5-substituted-2-amino-1,3,4-thiadiazoles were adequately prepared using ChemDraw Professional 16.0 software (licensed version) for the next in silico studies.

#### Molecular Docking Protocol

Blind docking of the selected three aminothiadiazoles, ChloViD2022, CoViTris2022, and Taroxaz-26, in SARS-CoV-2 RdRp and ExoN proteins was performed via MOE. Molnupiravir was used as a positive control anti-SARS-CoV-2 reference having proven potent RdRp/ExoN inhibitory activities. Prior to starting these docking procedures, some important preparations (mainly, additions and corrections) were required. All the missed atoms/residues in the SARS-CoV-2 RdRp and ExoN were added via MOE structure modeling. The two specific proteins were precisely prepared for molecular docking by the addition of hydrogen atoms using the 3D protonation module of the used MOE software; any scattered partial charges (which were internally originated due to the asymmetric distribution of chemical bonds electrons) were also corrected for both proteins. RdRp and ExoN were energy minimized in their complex forms via the Amber-99 force field which is available in MOE. Similarly, the structures of the three target ligands and molnupiravir were also adequately energy minimized in MOE. For docking of the target/reference ligands with the two proteins, the known London-dG scoring functions were utilized for binding energy calculations. For each docked target/reference molecule, the MOE software produced about twenty different poses with each docked SARS-CoV-2 protein. Of all the docking poses for each molecule with each protein, the one with the highest number of best molecular interactions, i.e., the top ranked pose of the best interactions, was recorded and saved. MOE gives a numerical value for the interaction of any potential ligand with any certain protein in the form of docking S-score (docking scores are expressed in kcal/mol). This docking binding energy or S-score represents the net energy of the formed protein–ligand complex and it also primarily reflects the degree of its expected stability (i.e., it provides a primary idea about the predicted stability of this formed complex prior to performing the more detailed robust computations via the molecular dynamics “MD” simulations). All the possible types of molecular interactions made during the docking processes were shown; these include, e.g., hydrogen bonding (H-bonds), hydrophobic interactions, ionic interactions/bonds, and salt bridges. For the four compounds, CoViTris2022, Taroxaz-26, ChloViD2022, and molnupiravir, respectively, the 2D and 3D output images of all the produced protein–ligand complexes (showing almost all the possible interactions) were saved for reporting and further investigative analysis.

#### Molecular Dynamics (MD) Simulation Protocol

The aforementioned three ligands ranked with the top results, e.g., with the best molecular interactions, lowest docking score (S-score), and lowest root-mean-square deviation (RMSD), computed through MOE and the apoenzyme against both proteins was then employed for further in silico studies, mainly the MD simulation studies, using Schrodinger’s Desmond module MD Simulation software. For MD simulation of the opted compounds and the reference drug, the best docking poses of these ligands in complexes with the SARS-CoV-2 RdRp and ExoN enzymes were kept in PDB format in MOE to be used for further virtual stability studies in Schrodinger’s Desmond module. The in-built Desmond System Builder tool was used in the current protocol to create the solvated water-soaked MD Simulation system. The TIP3P model was utilized as the solvating model in the present experiment. With periodic boundary conditions, an orthorhombic box was accurately simulated with a good boundary distance of at least 10 Å from the outer surface of each of the two coronaviral-2 proteins. The simulation systems were neutralized of complex charges by the addition of a reasonably sufficient amount of counter ions. The isosmotic state was maintained by adding 0.10 mol/l sodium and chloride ions, i.e., 0.10 M NaCl, into the simulation panel. Prior to beginning the simulation process, a predefined equilibration procedure was done. The system of the MD simulation was equilibrated by employing the standard Desmond protocol at a constant pressure of 1.0 bar and a constant temperature of 300 K (NPT ensemble; considering the viral nature of the two targeted enzymatic proteins) and also by employing the known Berendsen coupling protocol with one temperature group. H-bond length was properly constrained using the validated SHAKE algorithm. Particle Mesh Ewald (PME) summation method was used to specifically model long-range electrostatic interactions. On the other hand, an exact cut-off of 10 Å was specifically assigned for van der Waals and short-range electrostatic interactions. As previously mentioned, the MD simulation was run at ambient pressure conditions of about 1.013 bar, while the used temperature was exactly set to 300 K for each 100-nsec (ns) period of this MD simulation, and 1000 frames were saved into the simulation trajectory file. The simulation run time for each complex system and apo system was fixed to 100 ns as a total. Please note that the main endpoint (cut-off point) used for comparison was the 100-ns point, the point where almost the extreme interaction and positioning of each ligand in the active pocket of each protein were reached. We also gave special attention to the last simulation interval, i.e., the 80–100-ns period, due to its greater practical importance when comparing results. In addition, the most constant/balanced and best results in most cases were surprisingly obtained in this last interval. It is worth mentioning that the average results during the full 100-ns simulations were similarly put into our consideration during analyzing the final results. After simulations, the trajectory file of the simulated system used for calculation of the various structural parameters required, e.g., RMSD (Å), root-mean-square fluctuation (RMSF; Å), radius of gyration (rGyr; Å), number of protein–ligand contacts (# of total contacts), interactions fractions (%), intermolecular H-bonds (from all aspects), molecular surface area (MolSA; Å^2^), solvent-accessible surface area (SASA; Å^2^), and polar surface area (PSA; Å^2^), to extensively perform stability studies of the complex and apo systems. The results of the promising three target compounds along with those of the reference drug were saved to be reported, discussed, and compared in the present paper.

### In Vitro Biological Evaluation

#### Specifications of the Bioassayed Compounds

Samples of the target compounds, ChloViD2022, CoViTris2022, and Taroxaz-26, were obtained from our previous work (Purity of each of them: ≥ 95%) [23]. While the reference anti-COVID-19 drug molnupiravir (EIDD-2801, CAS Registry Number: 2349386-89-4) was purchased from Biosynth Carbosynth (Carbosynth Ltd., Berkshire, U.K.) (Product Code: AE176721, Purity: ≥ 98%). The ultrapure solvent dimethyl sulfoxide (DMSO, CAS Registry Number: 67-68-5) was purchased from a local distributor, El-Gomhouria Company For Drugs (El-Gomhouria Co. For Trading Drugs, Chemicals & Medical Supplies, Mansoura Branch, Egypt) (Purity: ≥ 99.9% “anhydrous”).

#### In Vitro Anti-RdRp/Anti-ExoN Assay (SARS-CoV-2-RdRp-Gluc Reporter Assay) of the Selected Aminothiadiazoles

First, the used cells, 293T cells (ATCC CRL-3216), were kept in Dulbecco’s modified Eagle’s medium (DMEM; Gibco) with 10% (v/v) fetal bovine serum (FBS; Gibco), then they were cultured at 37 °C in a humidified atmosphere of CO_2_ (5%). HEK293T cells were transfected using Vigofect transfection reagents (Vigorous) according to the strict instructions of the manufacturer. The required plasmid DNAs, antibodies, and reagents were purchased and treated exactly as in the literature procedures [25, 26]. The tested drugs are as described and specified in Subsection "Specifications of the Bioassayed Compounds". Also, western blotting (for the collected transfected HEK293T cells), real-time RT-PCR (for the extracted total RNA of transfected HEK293T cells), and cell viability test (using Cell Counting Kit-8 (CCK8), Beyotime) were exactly performed as the typical procedures of the literature [25, 26]. The steps of the well designed in vitro SARS-CoV-2-RdRp-Gluc Reporter Assay were accurately carried out according to the same original method of literature but with almost all the proteins modified and relevant to the SARS-CoV-2 Omicron variant “B.1.1.529.1/BA.1 sublineage” (HEK293T cells were transfected in this biochemical assay with CoV-Gluc, nsp12, nsp7, and nsp8 plasmid DNAs at the ratio of 1:10:30:30, and with CoV-Gluc, nsp12, nsp7, nsp8, nsp10, and nsp14 plasmid DNAs at the ratio of 1:10:30:30:10:90) [25, 26]. Exactly as instructed in the original assay, a stock of coelenterazine-h was dissolved in absolute ethanol (of very pure analytical grade) to a concentration of 1.022 mM [25, 26]. Directly before each assay, the stock was diluted in phosphate-buffered saline (PBS) to a concentration of 16.7 μM and incubated in the dark for 30 min at room temperature [25, 26]. For luminescence assay, 10 μl of supernatant was added to each well of a white and opaque 96-well plate, then 60 μl of 16.7 μM coelenterazine-h was injected, and luminescence was measured for 0.5 s using the Berthold Centro XS3 LB 960 microplate luminometer [25, 26]. Final results were statistically represented as the mean (*µ*) ± the standard deviation (*SD*) from at least three independent experiments. Statistical analysis was performed using SkanIt 4.0 Research Edition software (Thermo Fisher Scientific) and Prism V5 software (GraphPad). All resultant data were considered statistically significant at *p* < 0.05.

#### In Vitro Anti-SARS-CoV-2 and Cytotoxic Bioactivities Multiassay of the Selected Aminothiadiazoles

This validated in vitro anti-COVID-19 multiassay (including the cytotoxicity test), which was designed for the assessment of the net anti-SARS-CoV-2 activities of potential anti-COVID-19 agents, is based mainly upon the authentic procedures of Rabie [5, 13, 14, 16–19]. The complete procedures were carried out in a specialized biosafety level 3 (BSL-3) laboratory. The assayed new strain of SARS-CoV-2 virus, the Omicron variant, B.1.1.529.1/BA.1 sublineage, was isolated from the fresh nasopharynx aspirate and throat swab of a 59-year-old Sudanese woman with confirmed COVID-19 infection on March 21, 2022. Vero E6 cells (ATCC CRL-1586) were infected with the viral isolate. The starting titer of the stock virus (10^7.25^ TCID_50_/ml) was prepared after three serial passages in Vero E6 cells in infection media (DMEM supplemented with 4.5 g/l D-glucose, 100 mg/l sodium pyruvate, 2% FBS, 100,000 U/l Penicillin–Streptomycin, and 25 mM *N*-(2-hydroxyethyl)piperazine-*N*′-ethanesulfonic acid (HEPES)). The tested target and reference compounds are as described and specified in Subsection "Specifications of the Bioassayed Compounds". Preliminary pilot assays were performed mainly to determine the best concentration of the tested compounds to begin the in vitro anti-SARS-CoV-2 and cytotoxicity tests with. Accordingly, the stocks of the tested compounds were precisely prepared by dissolving each of the four compounds in DMSO to obtain a final concentration of 100 μM of each compound. Additionally, extrapure DMSO (100%) was used for the purpose of a negative control comparison to make this experimental study placebo controlled. To assess the total in vitro anti-SARS-CoV-2 activity of each of the target drugs, ChloViD2022, CoViTris2022, and Taroxaz-26, in comparison to that of the positive control/reference drug, molnupiravir, along with that of the negative control solvent, DMSO, Vero E6 cells were pretreated with each of the five compounds diluted in infection media for 1 h prior to infection by the new Omicron variant of the SARS-CoV-2 virus at MOI = 0.02. The tested five compounds were maintained with the virus inoculum during the 2-h incubation period. The inoculum was removed after incubation, and the cells were overlaid with infection media containing the diluted tested compounds (in serial concentrations). After 48 h of incubation at 37 °C, supernatants were immediately collected to quantify viral loads by TCID_50_ assay or quantitative real-time RT-PCR “qRT-PCR” (TaqMan Fast Virus 1-Step Master Mix). Viral loads in this assay were fitted in logarithm scale (log_10_ TCID_50_/ml, log_10_ TCID_90_/ml, and log_10_ viral RNA copies/ml), not in linear scale, under increasing concentrations of the tested compounds. Four-parameter logistic regression (GraphPad Prism) was used to fit the dose–response curves and determine the EC_50_ and EC_90_ of the tested compounds that inhibit SARS-CoV-2 viral replication (CPEIC_100_ was also determined for each compound). Cytotoxicity of each of the tested five compounds was also evaluated in Vero E6 cells using the CellTiter-Glo Luminescent Cell Viability Assay (Promega). Final results were statistically represented as the mean (*µ*) ± the standard deviation (*SD*) from at least three independent experiments. Statistical analysis was done using SkanIt 4.0 Research Edition software (Thermo Fisher Scientific) and Prism V5 software (GraphPad). All produced data were considered statistically significant at *p* < 0.05.

## Results and Discussion

### Computational Molecular Modeling of the Selected Aminothiadiazoles as Potential Anti-COVID-19 Drugs

After performing the exploratory computational screening and filtration of the small library of previously synthesized 5-substituted-2-amino-1,3,4-thiadiazoles (as mentioned in the Materials and Methods section), the top three compounds with the best and most ideal results of conventional pharmacodynamic/pharmacokinetic parameters with respect to the predicted anti-SARS-CoV-2 properties were selected for our sublime mission. The chosen compounds were, respectively, as follows: ChloViD2022, CoViTris2022, and Taroxaz-26. As previously mentioned, these three compounds are all synthetic molecules (Fig. 1A) and many of their typical analogs have demonstrated strong antiviral capabilities either in computational (predicted) or experimental (practical) studies or in both of them. This antiviral potential of their respective analogs is one of the main reasons we have examined these prospective inhibitors in the current virtual docking and simulation studies with SARS-CoV-2 RdRp and ExoN enzymes. Specific molecular docking against SARS-CoV-2 RdRp and ExoN revealed that the three target compounds ChloViD2022, CoViTris2022, and Taroxaz-26, respectively, have considerably low binding free energies (ranged from −6.0 to −7.9 kcal), which are significantly comparable to the reference anti-RdRp/anti-ExoN drug molnupiravir (having binding energies ranged from −6.7 to −7.1 kcal), as shown in Table 1. It was also predicted that ChloViD2022 and CoViTris2022 are, respectively, more promising target ligands than Taroxaz-26, exhibiting very good S-scores compared to the reference NA molnupiravir (this is the starting point of the current research). The catalytic pockets (i.e., active sites) of the two coronaviral-2 enzymes, RdRp (which is the main enzyme responsible for replication and transcription of the coronaviral-2 RNA genome) and ExoN (it is worth mentioning that nsp14 or the proofreading exoribonuclease of SARS-CoV-2 has two active sites; the exoribonuclease active site, the major one that we are concerned with in the current study, and the methyltransferase active site), were nearly detected and validated through previous several computational, crystallographic, and biochemical experiments in the literature [30–33]. Investigating and analyzing the resultant in silico interactions of the aforementioned three molecules with the residues of SARS-CoV-2 RdRp and ExoN proteins showed that all molecules significantly hit most of the active amino acid residues of the catalytic pockets of both enzymes with strong interactions (of gradual forces), including, mainly, H-bonds, hydrophobic interactions, ionic bonds, and water bridges, of comparatively short bond distances and low binding energies.

**Table 1:** The binding affinity energy values (docking S-scores) estimated during molecular docking of the screened three aminothiadiazoles, ChloViD2022, CoViTris2022, and Taroxaz-26, respectively, against the two SARS-CoV-2 proteins, RdRp and ExoN enzymes (using molnupiravir as the positive control drug)

Classification	Compound Name	Docking S-score (kcal/mol)	
		RdRp (7BV2)	ExoN (7MC6)
Screened Aminothiadiazoles	ChloViD2022	−7.1	−7.9
	CoViTris2022	−6.5	−6.8
	Taroxaz-26	−6.0	−6.6
Reference Drug	Molnupiravir	−6.7	−7.1

The three aminothiadiazoles are arranged in a collective descending order, beginning from the top ranked one and ending with the least ranked one

Figures S1–S4 in the Supplementary Material file show the detailed 2D and 3D representations of the most apparent intermolecular interactions between each ligand of the four ones (including the reference one) with each of the two coronaviral-2 enzymes, respectively. The 3D representations focus mostly on the shortest bonds. The molecules of the three target aminothiadiazoles strongly strike most of the neighboring active residues of the major catalytic pocket of SARS-CoV-2 RdRp (in chain A, i.e., 7BV2-A receptor), e.g., Arg553, Arg555, Cys622, Asp623, Arg624, Thr680, Ser681, Ser682, Thr687, Asn691, Leu758, Ser759, Asp760, and Asp761 (interactions with this last mentioned amino acid residue clearly appear in MD simulation outcomes later). On the other hand, the molecules of the same three aminothiadiazoles powerfully strike most of the adjacent active residues of the major catalytic pocket (exoribonuclease active site) of SARS-CoV-2 ExoN (in chain A; QHD43415_13 receptor), e.g., Asp90, Val91, Glu92, Gly93, His95, Asn104, Phe146, Trp186, Ala187, Phe190, Gln191, Asn252, Leu253, Gln254, His268, and Asp273 (interactions with the amino acid residues Gly93, His95, and Asn104 clearly appear in MD simulation outcomes later). Hypothetically at this point, these interactions are very promising and very comparable to, or even in some cases significantly better than, those of molnupiravir with the same two enzymes. Most of these time-independent findings were confirmed by the next MD simulation findings.

**Fig. 2:**
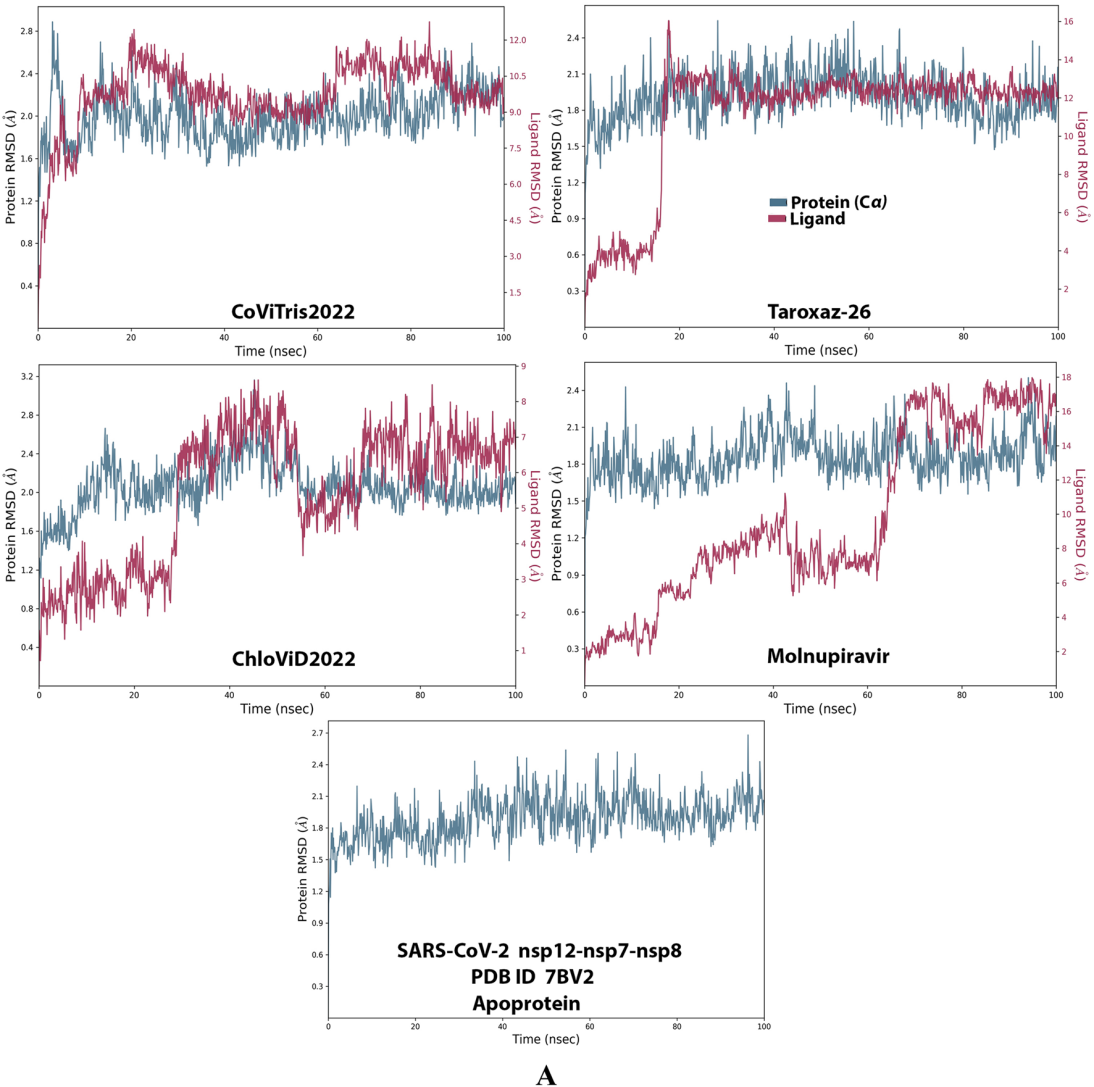

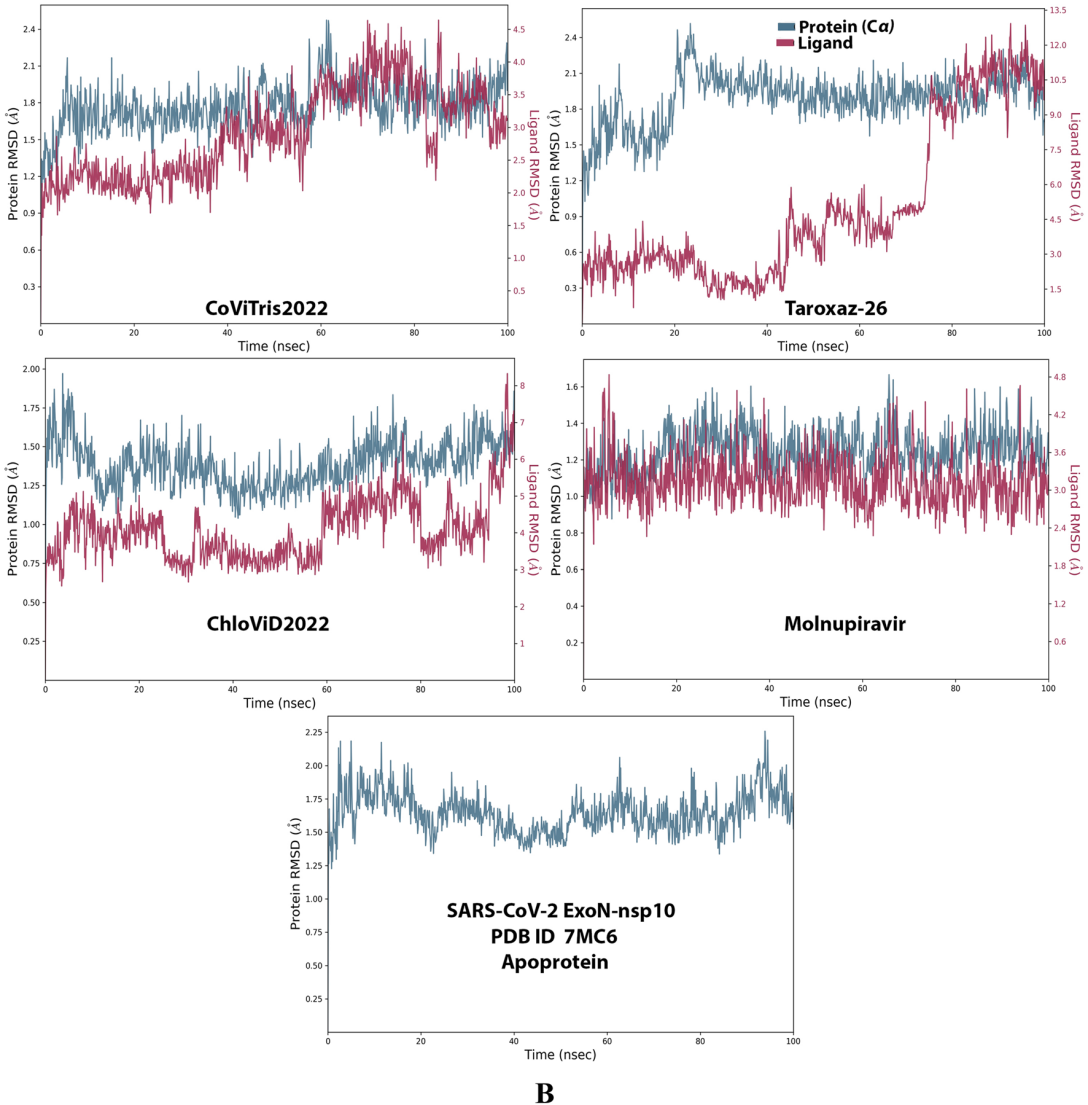
RMSD trajectories (during a simulation period of 100 ns) of the *α*-carbon of amino acid residues of the protein (blue color) and the ligand (maroon color) in the protein–ligand complexes of the three aminothiadiazoles, CoViTris2022, Taroxaz-26, and ChloViD2022, and the reference drug, molnupiravir, respectively, with: **A** SARS-CoV-2 RdRp “nsp12” enzyme cocrystallized with its protein cofactors nsp7 and nsp8 (PDB ID: 7BV2). **B** SARS-CoV-2 ExoN “nsp14” enzyme cocrystallized with its protein cofactor nsp10 (PDB ID: 7MC6). The apoprotein RMSD trajectories of the 7BV2 and 7MC6 were, respectively, displayed for comparison purposes

**Fig. 3:**
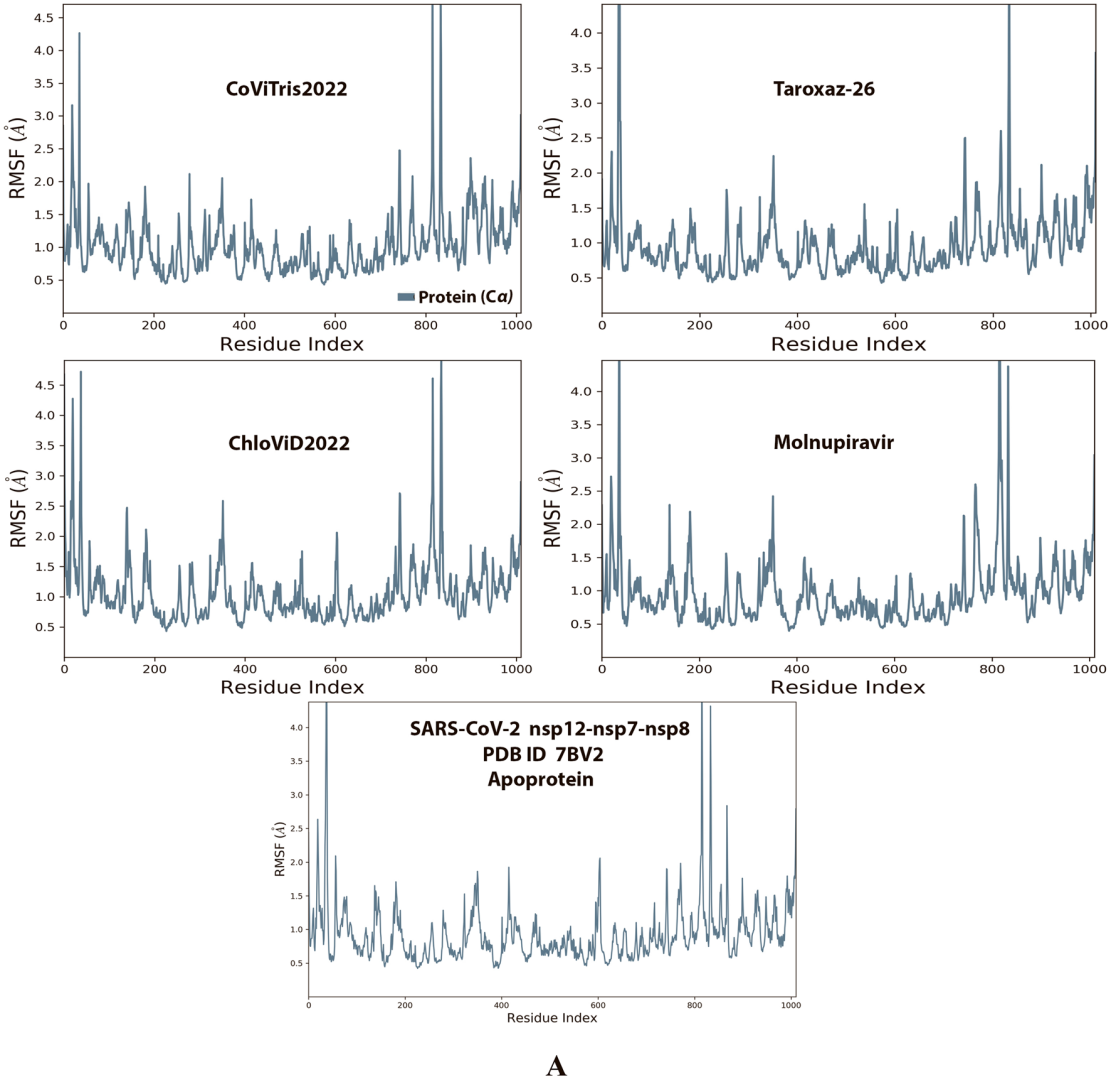

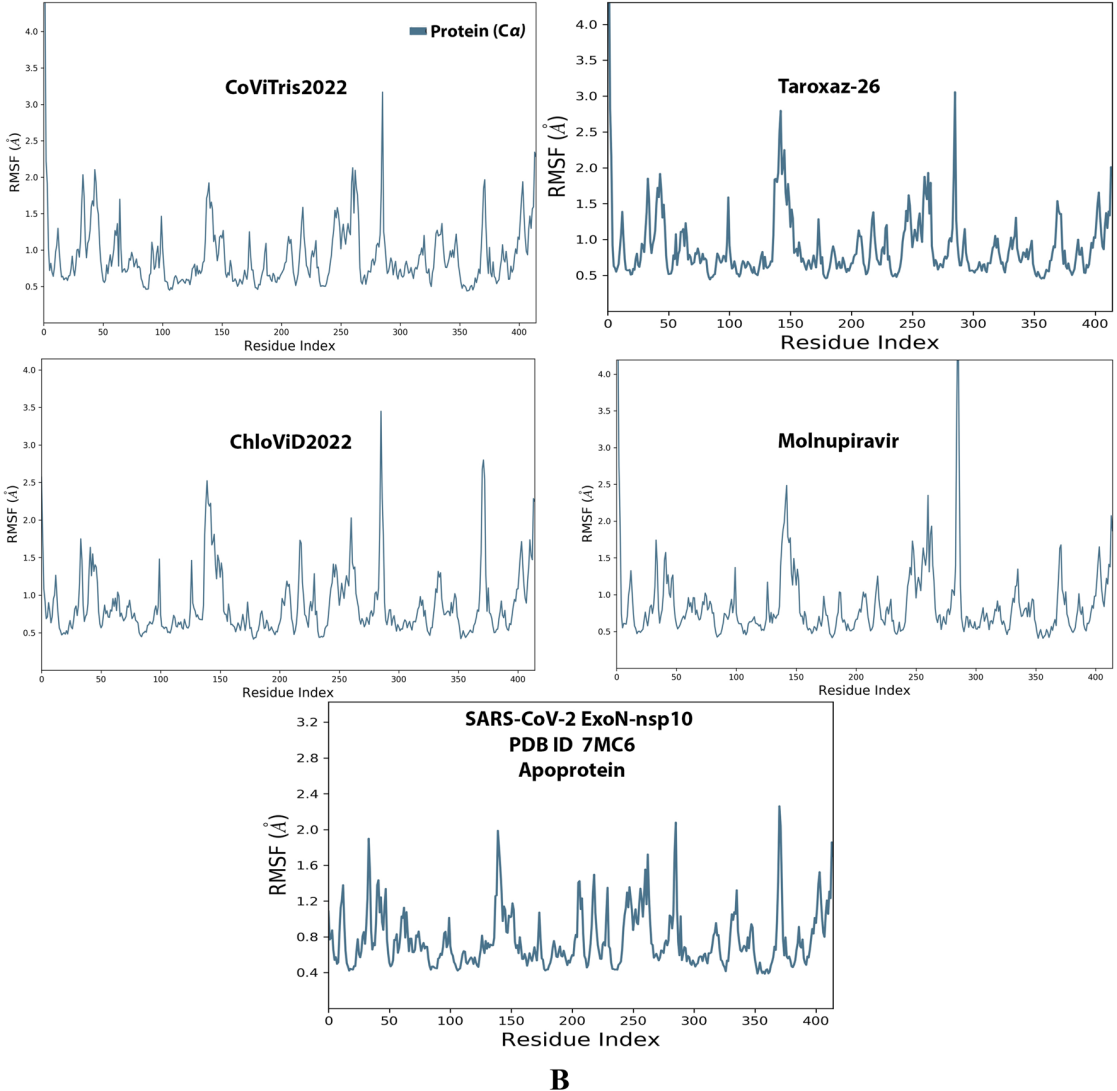
RMSF trajectories (along the different residue regions) of the *α*-carbon of amino acid residues of the protein in the protein–ligand complexes of the three aminothiadiazoles, CoViTris2022, Taroxaz-26, and ChloViD2022, and the reference drug, molnupiravir, respectively, with: **A** SARS-CoV-2 RdRp “nsp12” enzyme cocrystallized with its protein cofactors nsp7 and nsp8 (PDB ID: 7BV2). **B** SARS-CoV-2 ExoN “nsp14” enzyme cocrystallized with its protein cofactor nsp10 (PDB ID: 7MC6). The apoprotein RMSF trajectories of the 7BV2 and 7MC6 were, respectively, displayed for comparison purposes

**Fig. 4:**
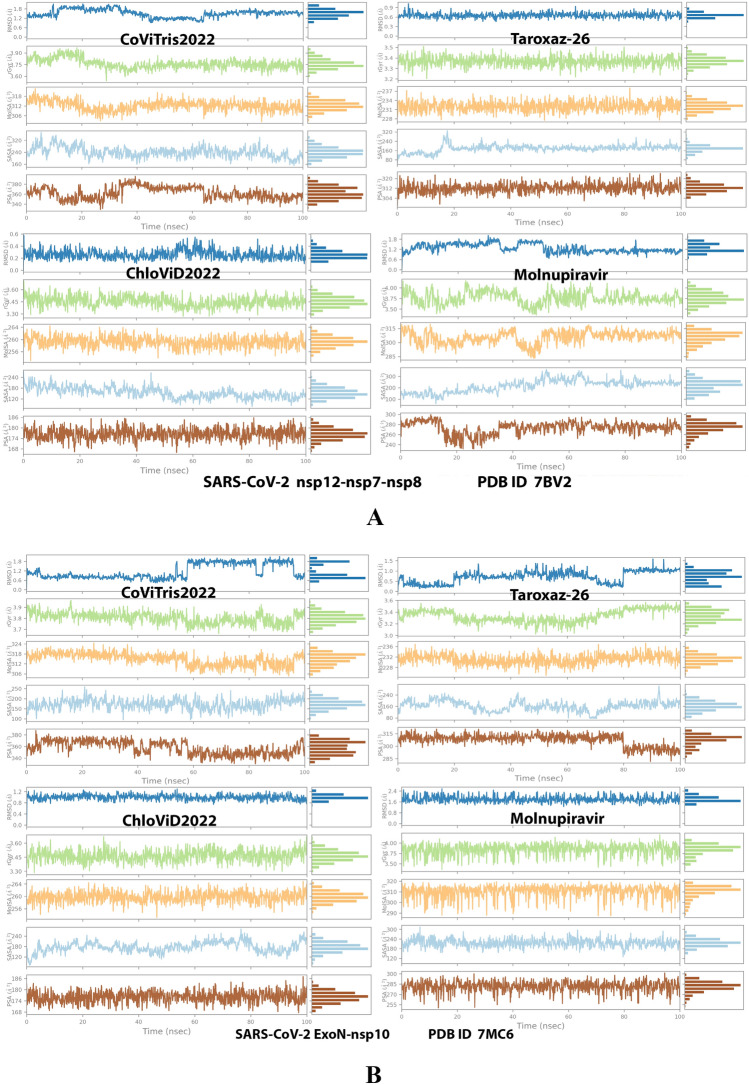
Collective post-MD simulation analysis of the protein–ligand complexes properties (RMSD, rGyr, MolSA, SASA, and PSA) of the three aminothiadiazoles, CoViTris2022, Taroxaz-26, and ChloViD2022, and the reference drug, molnupiravir, respectively, with: **A** SARS-CoV-2 RdRp “nsp12” enzyme cocrystallized with its protein cofactors nsp7 and nsp8 (PDB ID: 7BV2). **B** SARS-CoV-2 ExoN “nsp14” enzyme cocrystallized with its protein cofactor nsp10 (PDB ID: 7MC6)

**Fig. 5:**
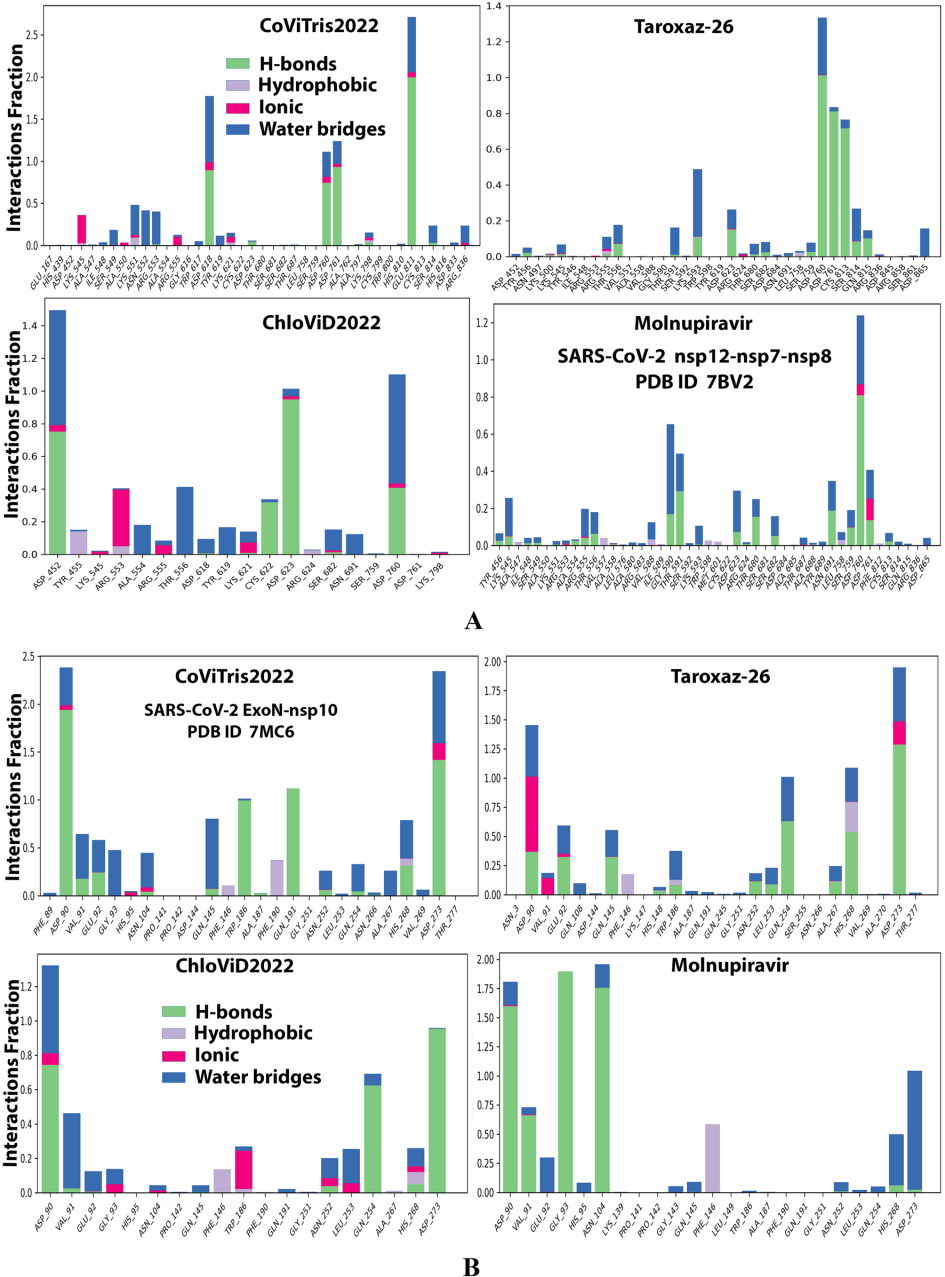
Histograms of fractions of the most predominant and stable protein–ligand interactions throughout the 100-ns simulative interaction trajectories of the three aminothiadiazoles, CoViTris2022, Taroxaz-26, and ChloViD2022, and the reference drug, molnupiravir, respectively, with: **A** SARS-CoV-2 RdRp “nsp12” enzyme cocrystallized with its protein cofactors nsp7 and nsp8 (PDB ID: 7BV2). **B** SARS-CoV-2 ExoN “nsp14” enzyme cocrystallized with its protein cofactor nsp10 (PDB ID: 7MC6)

**Fig. 6:**
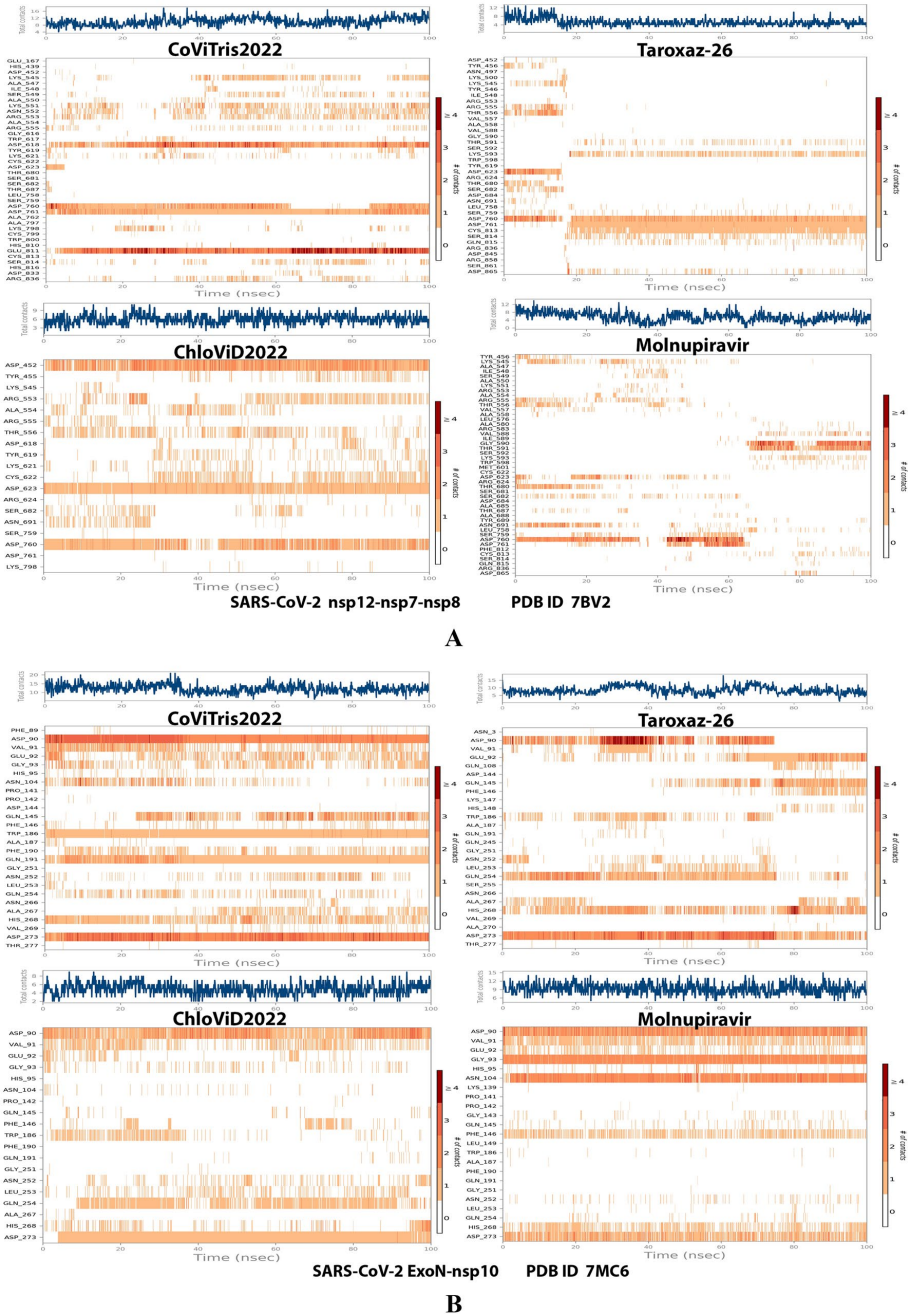
Plots of distribution of the total number of the most predominant and stable interactions (contacts) in each trajectory framework of the protein–ligand complexes of the three aminothiadiazoles, CoViTris2022, Taroxaz-26, and ChloViD2022, and the reference drug, molnupiravir, respectively, with: **A** SARS-CoV-2 RdRp “nsp12” enzyme cocrystallized with its protein cofactors nsp7 and nsp8 (PDB ID: 7BV2). **B** SARS-CoV-2 ExoN “nsp14” enzyme cocrystallized with its protein cofactor nsp10 (PDB ID: 7MC6)

Analysis of the MD simulation results revealed the relative stability of the formed protein–ligand complex of each of the three aminothiadiazoles with each of the two enzymes when compared with the reference drug. Also, comparing the kinetics and conformations of each enzyme in the ligand complex statuses with those in the original ligand-free (the control apoprotein) statuses showed very slight and tolerable modifications which approaching high stability at the endpoint in most cases. All comparisons were made with a major focus on the most productive (stable) region/phase of the simulation (almost the 80–100-ns period); however, the average values (throughout the entire simulation periods) and the endpoint values (at 100 ns) were also considered in the overall final analysis. Complexes of the three target compounds with SARS-CoV-2 ExoN are slightly more stable, with comparatively less numbers/intensities of fluctuations and with lower RMSD (Å) and RMSF (Å) values than those with SARS-CoV-2 RdRp. Interestingly, ChloViD2022 displayed superiority among all the three 1,3,4-thiadiazole derivatives in many of the compared MD items (including the best balanced RMSD and RMSF values in most cases) during the simulation. Comprehensively, the RdRp-ChloViD2022, RdRp-CoViTris2022, RdRp-Taroxaz-26, ExoN-ChloViD2022, ExoN-CoViTris2022, and ExoN-Taroxaz-26 complexes appear to be reasonably stable (i.e., stable with relatively acceptable degrees when compared with the corresponding complexes of the reference drug molnupiravir). The few early fluctuations (which were not mostly extreme) in RMSF and RMSD trajectories may be indications of some compulsory conformational changes within the enzymatic complex system as a result of the proper redirecting and repositioning of each ligand of the three target ones inside the catalytic binding sites which take some nanotime till the formation of very interesting strong molecular interactions. Possible unrevealed allosteric modulations, especially in case of the larger protein complex SARS-CoV-2 nsp12-nsp7-nsp8, could also be put into consideration. Taroxaz-26 and ChloViD2022 have the lowest rGyr values (mostly less than 3.6 Å) among all the tested compounds, including the reference molnupiravir (has rGyr value over 4.0), with both enzymes, indicating more compact and stable protein systems. In addition, from the computational point of view, ChloViD2022 followed by Taroxaz-26 have the best balanced MolSA, SASA, and PSA values among all the investigated four compounds. Interestingly, CoViTris2022 displayed the largest two interactions fractions (each of about 2% of the total interactions predicted) of the strong H-bonds with the hit SARS-CoV-2 proteins, among all the tested compounds, and this occurs specifically with the two catalytic amino acid residues Glu811 and Asp90 in the large protein SARS-CoV-2 nsp12-nsp7-nsp8 and the smaller protein SARS-CoV-2 nsp14-nsp10, respectively, in their relatively stable complexes with CoViTris2022 molecule, indicating significant potentials of CoViTris2022 to give strongly inhibited/blocked statuses of the RdRp and ExoN enzymes. MD simulation results also confirmed nearly all the primary molecular docking data with regard to, for example, the interacting amino acids along with the numbers/types/strengths of the formed bonds. Figures 2A and B, 3A and B, 4A and B, 5A and B, and 6A and B show the detailed results of MD simulation of the interactions between each ligand of the promising three aminothiadiazoles, CoViTris2022, Taroxaz-26, and ChloViD2022, with each of the two coronaviral-2 enzymes, RdRp and ExoN, respectively (in comparison with the reference FDA-approved anti-SARS-CoV-2 RdRp drug molnupiravir). The previous computational data were very encouraging to motivate us to transfer to the biological assessment part of the current work.

### Experimental Biological Evaluation of the Selected Aminothiadiazoles as Potential Anti-COVID-19 Drugs

The first preclinical assay in this extensive assessment is the robust cell-based test, the in vitro anti-SARS-CoV-2 RdRp (or, more accurately, the anti-SARS-CoV-2 RdRp/ExoN) bioassay, which was recently developed using Gaussia luciferase (Gluc) as the reporter to assess the anticoronaviral-2 RdRp activity of mainly the NAs (the prodrugs of nucleotides) without any necessity for generating the active nucleotidic triphosphate forms of the NAs (or of the other nontriphosphorylated nucleotidic analogs, i.e., of the monophosphorylated and diphosphorylated NAs) as for the cell-free assays [25, 26]. Furthermore, it was undoubtedly confirmed, through the findings of this new biochemical assay, that the exonuclease activity of SARS-CoV-2 nsp14 significantly improves the SARS-CoV-2 RdRp resistance to the various inhibitors of the nucleoside/nucleotide analogs class (one of the primary factors that aggravates the resistance and severe pathogenicity of SARS-CoV-2 particles is their abilities to encode the nsp14 ExoN which is capable of taking off the faulty mutagenic nucleotides misintegrated by the low-fidelity RdRp into the growing coronaviral-2 RNA strands, causing considerable resistance to nucleos(t)ide analog therapeutic agents), thus ExoN effects were considered and added in the steps of this screening assay protocol which was primarily designed for exploring possible SARS-CoV-2 RdRp inhibitors (dissimilar to the traditional analytical cell-free assay) [25, 26, 34, 35].

As previously mentioned, we mainly concentrate here on the two principal protein complexes that catalyze and control the SARS-CoV-2 replication/transcription processes, nsp12-nsp7-nsp8 polymerase complex and nsp14-nsp10 exoribonuclease complex, respectively. This test significantly simulates the respective original replication processes that occur for the SARS-CoV-2 genome, as it functionally mimics the RNA generating processes driven mainly by the SARS-CoV-2 RdRp [36]. Table 2 displays the detailed values obtained from this in vitro anti-SARS-CoV-2 RdRp/ExoN bioassay. The resultant data showed that, among the tested three target aminothiadiazoles, the nucleoside-like compound ChloViD2022 demonstrated the best results. However, the three compounds effectively inhibited SARS-CoV-2 RdRp activity with significantly low EC_50_ values of 0.17, 0.21, and 0.23 μM, which slightly increased in the presence of SARS-CoV-2 ExoN (the wild type) to about 0.27, 0.33, and 0.39 μM, respectively, indicating the potent inhibitory/blocking activities of the three compounds against SARS-CoV-2 ExoN (see the extremely minute nanomolar differences of the EC_50_ values between both cases). Mutations in the exoribonuclease (i.e., the mutated type; e.g., D90A/E92A mutations of the active catalytic residues in nsp14 as in our current case) reinforced the anti-RdRp activity of ChloViD2022, CoViTris2022, and Taroxaz-26 to excellent EC_50_ values of 0.22, 0.26, and 0.31 μM (i.e., slightly lower than that resulted in the presence of the normal wild type of ExoN; these very slight changes also reflected, as previously mentioned, the potent activities of the three target aminothiadiazoles against SARS-CoV-2 ExoN in its original wild type from the beginning prior to any intended mutations). These previous values of ChloViD2022, CoViTris2022, and Taroxaz-26, respectively, even beat those of the potent reference anti-SARS-CoV-2 agent, molnupiravir, which showed higher values, reflecting the prospective superiority of the three NAs over molnupiravir in clinical investigation in humans. The results also proved that molnupiravir could not resist the performance of Omicron-variant ExoN the same way and potency ChloViD2022, CoViTris2022, and Taroxaz-26 do. It is apparently observed from the values in Table 2 that as much the EC_50_ values of the NA against the polymerase alone and against the polymerase in the presence of the exoribonuclease are close to each other, as more potent this NA inhibitor is (i.e., as more predicted for this tested NA to be an ideally effective anti-RdRp or, more accurately, anti-SARS-CoV-2 replication). From the results we can also conclude that an ideal potent SARS-CoV-2 RdRp inhibitor should have a ratio of EC_50*(polymerase* + *exoribonuclease)*_/EC_50*(polymerase)*_ that is very close to 1 and less than 2. As this ratio decreases, as the compound has higher potentials to succeed in inhibiting the SARS-CoV-2 replication more efficiently. ChloViD2022 displayed the highest resistance, among all the tested compounds, to the coronaviral-2 nsp14 exoribonuclease activity in HEK293T cells. The very promising capabilities of ChloViD2022, CoViTris2022, and Taroxaz-26 to inhibit the nsp12 polymerase and nsp14 exoribonuclease activities of the coronaviral-2 Omicron variant interestingly uphold the repurposing potentials of ChloViD2022, CoViTris2022, and Taroxaz-26 in clinical settings for further therapeutic use as potent anti-COVID-19 drugs. It is worth mentioning that ChloViD2022, CoViTris2022, and Taroxaz-26 are nearly the only synthetic NAs of the 2-amino-1,3,4-thiadiazole type that have such unique potent anti-SARS-CoV-2 activities against both the RdRp and ExoN enzymes of the newest SARS-CoV-2 variant, Omicron variant, in very significant values to date (this is to the best of our current knowledge during the submission of this research paper for publication) [25, 26]. These present biochemical findings concerning the potent inhibitory SARS-CoV-2 RdRp-binding and ExoN-binding properties of ChloViD2022, CoViTris2022, and Taroxaz-26 are in an ideal agreement with almost all the computed parameters of the prior in silico part of this comprehensive research, which was discussed in details in Subsection "Computational Molecular Modeling of the Selected Aminothiadiazoles as Potential Anti-COVID-19 Drugs".

**Table 2:** Anti-SARS-CoV-2 RdRp/ExoN activities (along with respective ratios) of the target synthetic compounds ChloViD2022, CoViTris2022, and Taroxaz-26 (using molnupiravir as the positive control/reference drug and DMSO as the negative control/placebo drug), respectively, in HEK293T cells, expressed as EC_50_ values in μM

Classification	Compound Name	Inhibition of SARS-CoV-2 RdRp in vitro (EC_50_ in μM)^a^			Respective Ratios of EC_50_	
		Nsp12	Nsp12 + Nsp14	Nsp12 + Nsp14_mutant_	(Nsp12 + Nsp14)/Nsp12	(Nsp12 + Nsp14_mutant_)/Nsp12
Target Aminothiadiazoles	ChloViD2022	0.17 ± 0.02	0.27 ± 0.03	0.22 ± 0.02	1.59	1.29
	CoViTris2022	0.21 ± 0.03	0.33 ± 0.04	0.26 ± 0.03	1.57	1.24
	Taroxaz-26	0.23 ± 0.03	0.39 ± 0.05	0.31 ± 0.05	1.70	1.35
Reference Drug	Molnupiravir	0.24 ± 0.04	0.45 ± 0.05	0.34 ± 0.04	1.88	1.42
Placebo Solvent	DMSO	> 100	> 100	> 100	N.A.^b^	N.A.

Please note that, in this table, nsp12 refers to nsp12/7/8 complex, nsp14 refers to nsp14/10 complex, and nsp14_mutant_ refers to nsp14_mutant_/10 complex

^a^EC_50_ or 50% effective concentration is the concentration of the tested compound that is required for 50% reduction in the COVID-19 polymerase (SARS-CoV-2 RdRp) activity in vitro. EC_50_ is expressed in μM

^b^N.A. means not available (i.e., it was not determined)

The second assay is the collective in vitro anti-SARS-CoV-2 and cytotoxicity tests. Table 3 shows the resultant values from both tests in details. The used SARS-CoV-2 strain in the anticoronaviral-2 assay is the new variant of SARS-CoV-2, the Omicron variant B.1.1.529.1/BA.1 sublineage, which is one of the most infectious and resistant strains of the virus. The data displayed in the table interestingly revealed the significantly higher antiviral efficacies of each of the three NAs ChloViD2022, CoViTris2022, and Taroxaz-26 against the newly appeared variants of SARS-CoV-2 as compared to those of the positive control reference drug molnupiravir (the placebo drug DMSO showed extremely weak activities, i.e., negligible results). ChloViD2022, CoViTris2022, and Taroxaz-26 were found to efficiently inhibit and impair the entire SARS-CoV-2 replication/transcription in Vero E6 cells with EC_50_ values extremely smaller than the 100 μM value of stock concentration, continuing their superiorities over the reference NA molnupiravir exactly as in the previous anti-RdRp/ExoN biochemical assay. Promisingly, ChloViD2022 was proved to be very leading (i.e., ranked first among all the tested compounds) in its total anti-Omicron activity (EC_50_ = 0.41 μM), which was found to be about 6.4 times as effective as the reference drug molnupiravir (EC_50_ = 2.61 μM) with respect to the tested in vitro anti-B.1.1.529.1/BA.1/anti-SARS-CoV-2 activity. While CoViTris2022 and Taroxaz-26 were ranked second and third, respectively, among all the tested four compounds, in their total anti-Omicron activities (EC_50_ = 0.69 and 0.73 μM, respectively), which were found to be about 3.8 and 3.6 times, respectively, as effective as molnupiravir (ranked fourth) with respect to the same evaluated activity. According to the current cytotoxicity assay, the in vitro CC_50_ values of ChloViD2022, CoViTris2022, and Taroxaz-26, respectively, are significantly greater than 100 μM, thus these three synthetic 2,5-disubstituted-1,3,4-thiadiazoles are expected to have very advantageous high corresponding clinical selectivity indices “SIs” (SI_ChloViD2022_ > 243.9, SI_CoViTris2022_ > 144.9, and SI_Taroxaz-26_ > 137; while molnupiravir has narrower SI, SI_molnupiravir_ > 38.3), reflecting the specific/selective anti-RNA actions of the ChloViD2022, CoViTris2022, and Taroxaz-26 molecules against the new coronaviral-2 Omicron genome rather than the human genome. Moreover, ChloViD2022, CoViTris2022, and Taroxaz-26 displayed significantly small values of the concentration that results in 100% in vitro inhibition of the coronaviral-2 Omicron variant cytopathic effects (CPEIC_100_ = 1.09, 1,60, and 1.83 μM, respectively), which are less than the corresponding value of molnupiravir (CPEIC_100_ = 6.25 μM). In line with their potent activities against the infectious coronaviral-2 B.1.1.529.1/BA.1 strain, ChloViD2022, CoViTris2022, and Taroxaz-26 also showed very slight values of the concentration that is needed for 50% in vitro lowering in the number of RNA copies of the B.1.1.529.1/BA.1 strain of SARS-CoV-2 (0.44, 0.70, and 0.77 μM, respectively), which are apparently smaller than the corresponding value of molnupiravir (2.73 μM). EC_90_ values of ChloViD2022, CoViTris2022, and Taroxaz-26, which are preferably used for the in vivo/clinical studies, were also very small (much smaller than that of molnupiravir) and consistent with the EC_50_ values (being not far that much from the EC_50_ values indicates the expected significant clinical potencies of the three compounds), as demonstrated in Table 3.

**Table 3:** Anti-SARS-CoV-2/anti-COVID-19 activities (along with cytotoxicities) of the target synthetic compounds ChloViD2022, CoViTris2022, and Taroxaz-26 (using molnupiravir as the positive control/reference drug and DMSO as the negative control/placebo drug), respectively, against SARS-CoV-2 (Omicron variant, B.1.1.529.1/BA.1 sublineage) in Vero E6 cells

Classification	Compound Name	CC_50_^a^ (μM)	Inhibition of SARS-CoV-2 Replication in vitro (Anti-B.1.1.529.1/BA.1 Bioactivities) (μM)			
			100% CPE Inhibitory Concentration (CPEIC_100_)^b^	50% Reduction in Infectious Virus (EC_50_)^c^	50% Reduction in Viral RNA Copy (EC_50_)^d^	90% Reduction in Infectious Virus (EC_90_)^e^
Target Aminothiadiazoles	ChloViD2022	> 100	1.09 ± 0.04	0.41 ± 0.02	0.44 ± 0.02	1.53 ± 0.05
	CoViTris2022	> 100	1.60 ± 0.06	0.69 ± 0.03	0.70 ± 0.04	2.01 ± 0.07
	Taroxaz-26	> 100	1.83 ± 0.07	0.73 ± 0.03	0.77 ± 0.04	2.34 ± 0.08
Reference Drug	Molnupiravir	> 100	6.25 ± 0.31	2.61 ± 0.10	2.73 ± 0.11	9.16 ± 0.37
Placebo Solvent	DMSO	> 100	> 100	> 100	> 100	> 100

^a^CC_50_ or 50% cytotoxic concentration is the concentration of the tested compound that kills half the cells in an uninfected cell culture. CC_50_ was determined with serially diluted compounds in Vero E6 cells at 48-h postincubation using CellTiter-Glo Luminescent Cell Viability Assay (Promega)

^b^CPEIC_100_ or 100% CPE inhibitory concentration is the lowest concentration of the tested compound that causes 100% inhibition of the cytopathic effects (CPE) of SARS-CoV-2 B.1.1.529.1/BA.1 virus in Vero E6 cells under increasing concentrations of the tested compound at 48-h postinfection. Compounds were serially diluted from 100 μM concentration

^c^EC_50_ or 50% effective concentration is the concentration of the tested compound that is required for 50% reduction in infectious SARS-CoV-2 B.1.1.529.1/BA.1 virus particles in vitro. EC_50_ is determined by infectious virus yield in culture supernatant at 48-h postinfection (log_10_ TCID_50_/ml)

^d^EC_50_ or 50% effective concentration is the concentration of the tested compound that is required for 50% reduction in SARS-CoV-2 B.1.1.529.1/BA.1 viral RNA copies in vitro. EC_50_ is determined by viral RNA copies number in culture supernatant at 48-h postinfection (log_10_ RNA copies/ml)

^e^EC_90_ or 90% effective concentration is the concentration of the tested compound that is required for 90% reduction in infectious SARS-CoV-2 B.1.1.529.1/BA.1 virus particles in vitro. EC_90_ is determined by infectious virus yield in culture supernatant at 48-h postinfection (log_10_ TCID_90_/ml)

It was surprisingly observed that ChloViD2022, CoViTris2022, and Taroxaz-26 successfully act against the SARS-CoV-2 in a relatively rapid mode of action (i.e., a relatively rapid onset of action), with their maximal effectiveness against the Omicron-variant particles reached within about 4–10 h of starting administration and treatment. The possible phosphate esters of ChloViD2022, CoViTris2022, and Taroxaz-26 are expected to be as effective as the administered original forms or even much more (due to the predicted higher bioavailability and biocompatibility). The current results of this reliable bioassay are in excellent proper agreement with almost all the findings of the previous anti-RdRp biochemical assay along with the previous computational study (which was discussed in details in Subsection "Computational Molecular Modeling of the Selected Aminothiadiazoles as Potential Anti-COVID-19 Drugs") of the current comprehensive research.

## Conclusions and Future Therapeutic Applications

Recently, synthetic and/or natural NAs, PPhs, and 2-amino-1,3,4-thiadiazoles antivirals topped the scene as first and early choices for COVID-19 therapy [16–20, 37, 38]. The current comprehensive in silico/in vitro preclinical research study disclosed the anti-COVID-19 potentials of the three nucleoside-like 5-substituted-2-amino-1,3,4-thiadiazoles, ChloViD2022, CoViTris2022, and Taroxaz-26, with ChloViD2022 being the most promising potent SARS-CoV-2 RNA mutagen or, at least, the most promising coronaviral-2 replication inhibitor in general. The three 1,3,4-thiadiazoles are synthetic antioxidant agents that were previously synthesized, and their 2-mercapto derivatives were evaluated as good free radical scavengers, in one of our previous papers [23]. Physically, ChloViD2022, CoViTris2022, and Taroxaz-26 molecules have very flexible chemical structures that can easily tolerate chemical modifications in biosystems. It was clearly found in the current research study that coronaviral-2 particles are very sensitive to the three compounds and thoroughly mutated/inhibited by them. Interestingly, it was discovered that ChloViD2022, CoViTris2022, and Taroxaz-26 may effectively stop SARS-CoV-2 spreadability and pathogenicity (and, consequently, end COVID-19 infection as a whole) in the human body, mainly through severely hindering SARS-CoV-2 replication via a synergistic dual inhibitory mode of action against the two SARS-CoV-2 enzymes RdRp and ExoN. This double mode of action could be extended to a triple one if the expected inhibitory effects of the three drugs against kinases, especially on ADK, are extensively explored and proved in a next work. In addition, we confirmed that these three molecules do not act as Pan-Assay Interference Compounds (PAINS) through using a specialized web server called PAINS-Remover server (by running the False Positive Remover tool) [39, 40]. All the three target compounds (as well as the reference compound) successfully passed the rigorous filter of this server, thus the possibilities to cause false-positive assay readouts were excluded. Among the three 1,3,4-thiadiazole derivatives, specifically ChloViD2022 molecule could be seen as a typical NA or, more precisely, a typical dideoxynucleoside analog. Based on the current research observations, the three nucleoside-like 2-amino-1,3,4-thiadiazole compounds, ChloViD2022, CoViTris2022, and Taroxaz-26, are specifically prioritized as prospective COVID-19 therapeutic drugs (with very promising anti-SARS-CoV-2 EC_50_ values of 0.41, 0.69, and 0.73 μM, respectively, against the Omicron variant) and they generally warrant deeper pharmacological and clinical investigations to clearly understand their accurate therapeutic values as potential anti-SARS-CoV-2 agents.

### Supplementary Information

Below is the link to the electronic supplementary material.

Supplementary file1 (DOCX 11981 KB)
