# Supplementary material for: Potent Dual Polymerase/Exonuclease Inhibitory Activities of Antioxidant Aminothiadiazoles Against the COVID-19 Omicron Virus: A Promising In Silico/In Vitro Repositioning Research Study
Source: Mol Biotechnol. 2023 Jan 24;66(4):592–611. doi: 10.1007/s12033-022-00551-8 (PMC9870775; doi:10.1007/s12033-022-00551-8)
Supplement: Supplementary file 1 — Supplementary file1 (DOCX 11981 KB) [file 12033_2022_551_MOESM1_ESM.docx]

# *Supplementary Material*

# Potent Dual Polymerase/Exonuclease Inhibitory Activities of Antioxidant Aminothiadiazoles Against the COVID-19 Omicron Virus: A Promising In Silico/In Vitro Repositioning Research Study

***Authors' Names & Affiliations*:**

**Amgad M. Rabie^a,b,*^ and Wafa A. Eltayb^c,**^**

**^a^ Dr. Amgad Rabie's Research Lab. for Drug Discovery (DARLD), Mansoura City 35511, Mansoura, Dakahlia Governorate, Egypt**

**^b^ Head of Drug Discovery & Clinical Research Department, Dikernis General Hospital (DGH), Magliss El-Madina Street, Dikernis City 35744, Dikernis, Dakahlia Governorate, Egypt**

**^c^ Biotechnology Department, Faculty of Science and Technology, Shendi University, Shendi, Nher Anile, Sudan**

*** *Principal Corresponding Author*:**

**Dr. Amgad M. Rabie**

**E-mails:** [**amgadpharmacist1@yahoo.com**](mailto:amgadpharmacist1@yahoo.com)**,** [**dr.amgadrabie@gmail.com**](mailto:dr.amgadrabie@gmail.com)

**ORCID iD: 0000-0003-3681-114X**

**Postal Address: Dr. Amgad M. Rabie, 16 Magliss El-Madina Street, Dikernis City 35744, Dikernis, Dakahlia Governorate, Egypt**

**Mobile No.: 002-01019733188 & 002-01112900494 (Egypt)**

**** *Second Corresponding Author*:**

**Dr. Wafa A. Eltayb**

**E-mails:** [**wafa.ali.11338@gmail.com**](mailto:wafa.ali.11338@gmail.com)

**ORCID iD: 0000-0002-3981-201X**

**
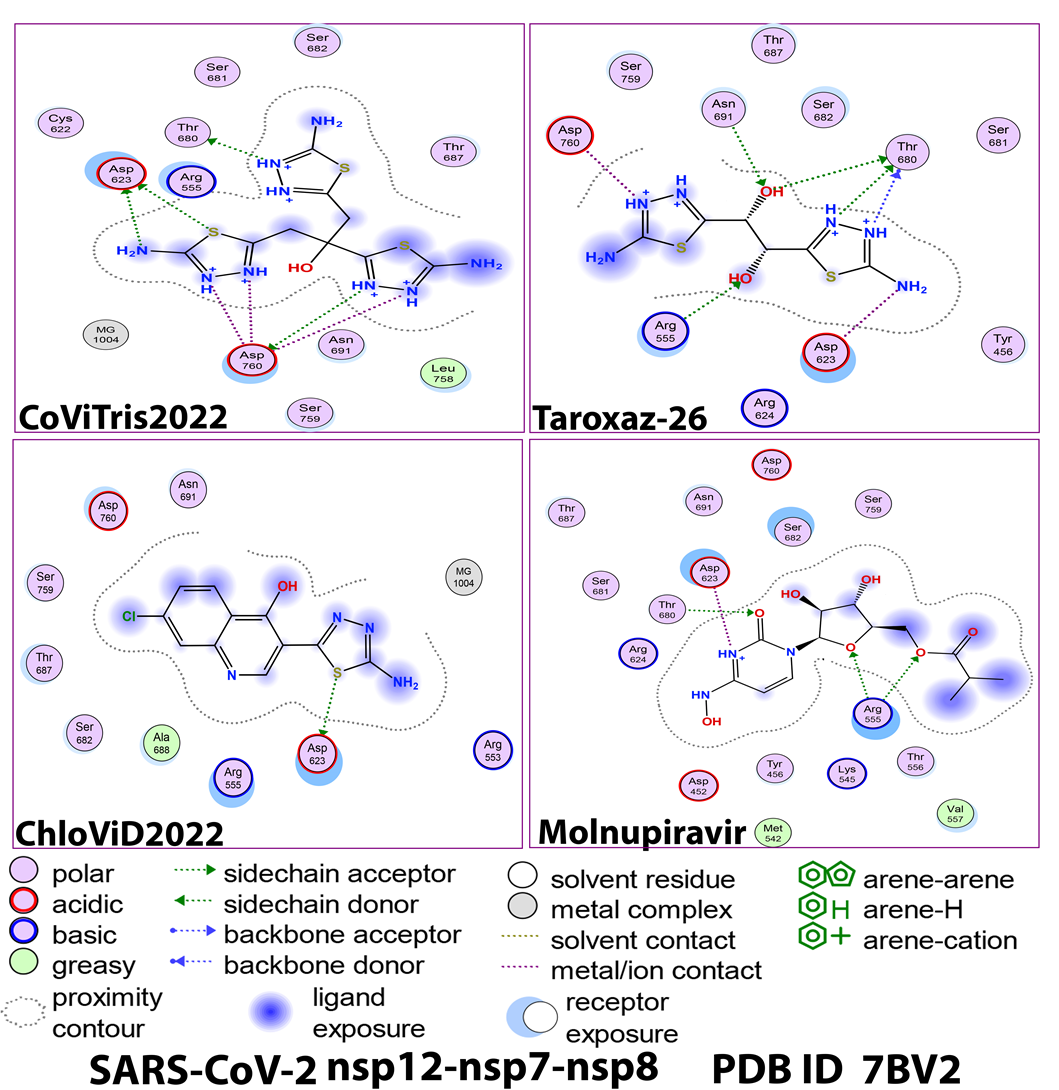
**

**Fig. S1.** 2D images of the postdocking interactions of the three aminothiadiazoles, CoViTris2022, Taroxaz-26, and ChloViD2022, and the reference drug, molnupiravir, respectively, with the SARS-CoV-2 RdRp "nsp12" enzyme cocrystallized with its protein cofactors nsp7 and nsp8 (PDB ID: 7BV2).


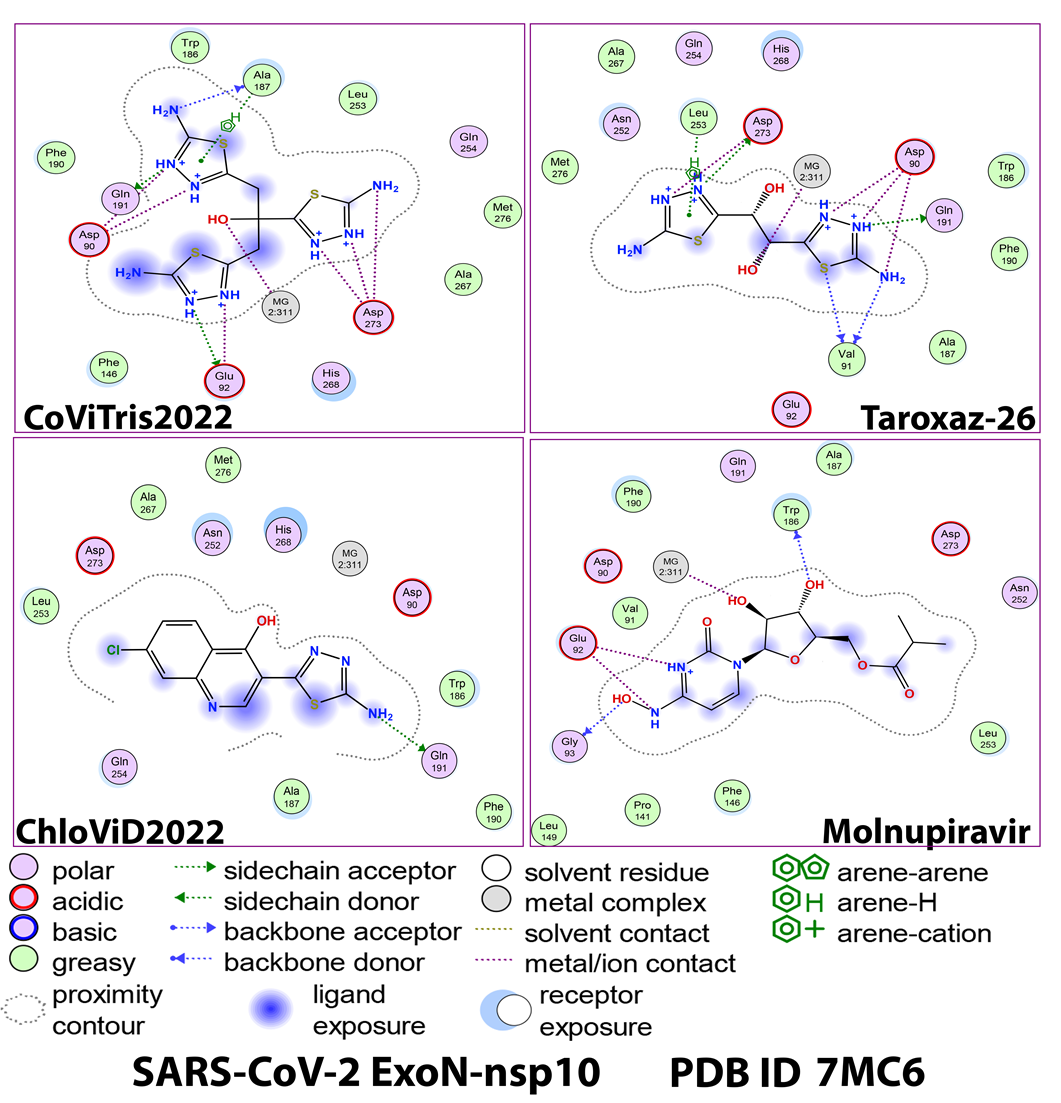


**Fig. S2.** 2D images of the postdocking interactions of the three aminothiadiazoles, CoViTris2022, Taroxaz-26, and ChloViD2022, and the reference drug, molnupiravir, respectively, with the SARS-CoV-2 ExoN "nsp14" enzyme cocrystallized with its protein cofactor nsp10 (PDB ID: 7MC6).

**
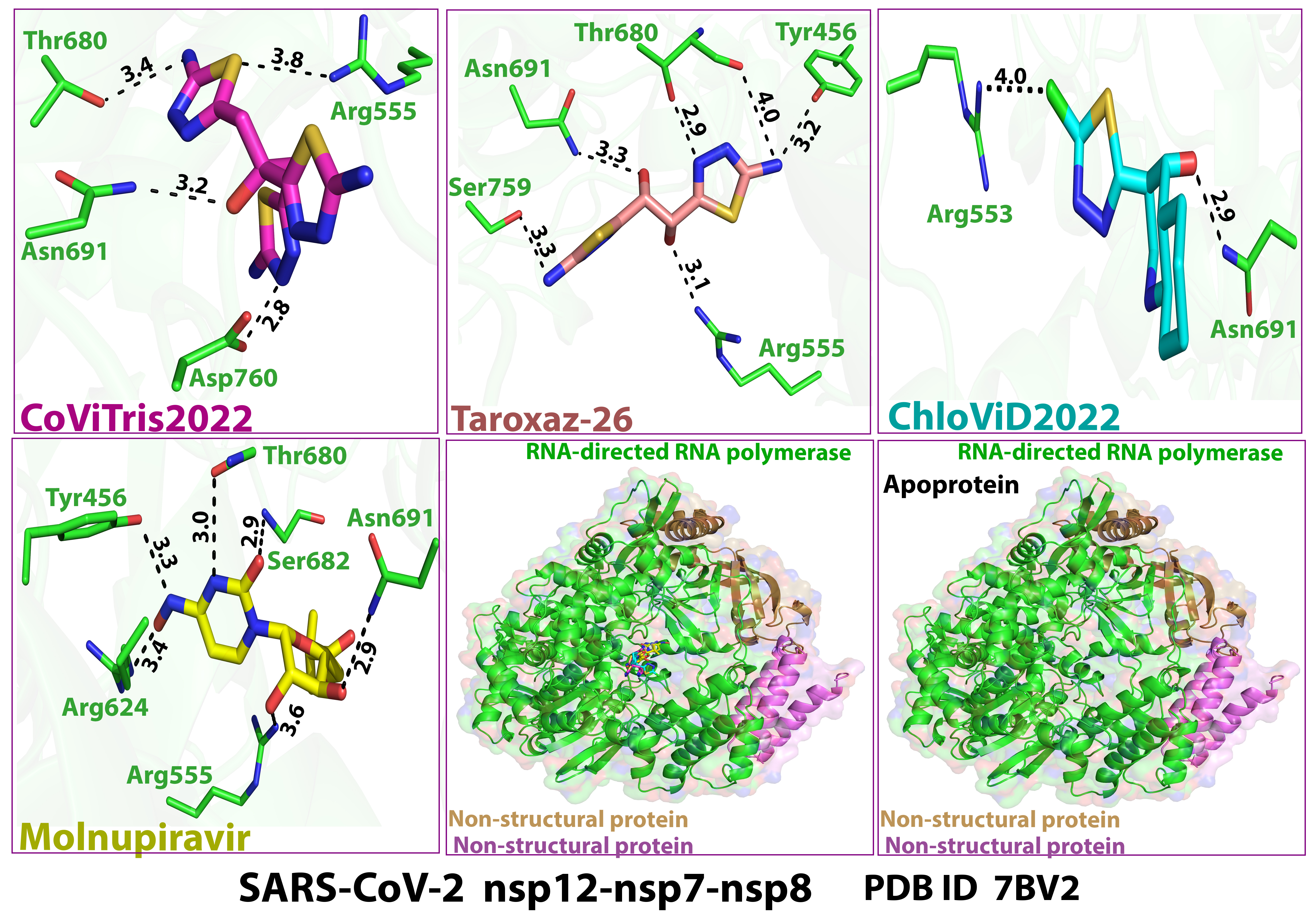
**

**Fig. S3.** 3D images of the postdocking interactions (showing the bond distances) of the three aminothiadiazoles, CoViTris2022, Taroxaz-26, and ChloViD2022, and the reference drug, molnupiravir, respectively, with the SARS-CoV-2 RdRp "nsp12" enzyme cocrystallized with its protein cofactors nsp7 and nsp8 (PDB ID: 7BV2).


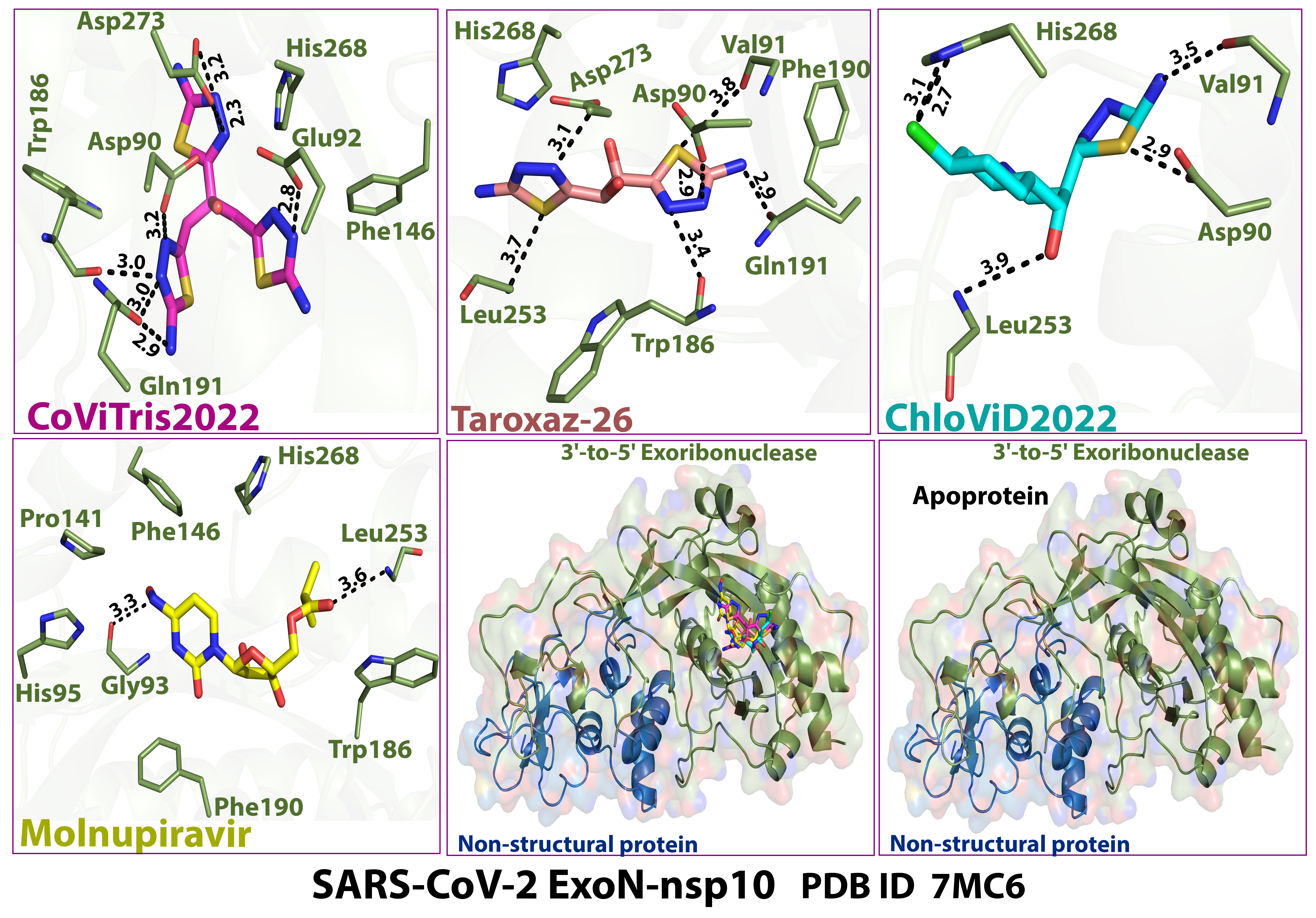


**Fig. S4.** 3D images of the postdocking interactions (showing the bond distances) of the three aminothiadiazoles, CoViTris2022, Taroxaz-26, and ChloViD2022, and the reference drug, molnupiravir, respectively, with the SARS-CoV-2 ExoN "nsp14" enzyme cocrystallized with its protein cofactor nsp10 (PDB ID: 7MC6).
